# Supplementary material for: Effects of supplemental lighting with different light qualities on growth and secondary metabolite content of Anoectochilus roxburghii
Source: PeerJ. 2018 Jul 19;6:e5274. doi: 10.7717/peerj.5274 (PMC6054866; doi:10.7717/peerj.5274)
Supplement: Supplemental Information 2 [file peerj-06-5274-s002.docx]

**1. The ANOVA of Leaf Numbers**

| **ANOVA** | | | | | |
| --- | --- | --- | --- | --- | --- |
| Leaf Numbers | | | | | |
|  | Sum of squares | df | Mean square | F | Sig. |
| Between Groups | 5.037 | 5 | 1.007 | 1.769 | .137 |
| Within groups | 27.333 | 48 | .569 |  |  |
| Total | 32.370 | 53 |  |  |  |

| **Multiple Comparisons** | | | | | | | | | |
| --- | --- | --- | --- | --- | --- | --- | --- | --- | --- |
| Dependent Variable: Leaf Numbers | | | | | | | | | |
|  | (I) Light Quality | | (J)  Light Quality | | Mean Difference (I-J) | Std.Errer | Sig. | 95% Confidence Internal | |
|  |  |  |  |  |  |  |  | lower Bound | Upper Bound |
| LSD | dimension2 | RL | dimension3 | BL | -.6667 | .3557 | .067 | -1.382 | .049 |
|  |  |  |  | YL | .2222 | .3557 | .535 | -.493 | .937 |
|  |  |  |  | GL | .1111 | .3557 | .756 | -.604 | .826 |
|  |  |  |  | WL | -.1111 | .3557 | .756 | -.826 | .604 |
|  |  |  |  | CK | .2222 | .3557 | .535 | -.493 | .937 |
|  |  | BL | dimension3 | RL | .6667 | .3557 | .067 | -.049 | 1.382 |
|  |  |  |  | YL | .8889^*^ | .3557 | .016 | .174 | 1.604 |
|  |  |  |  | GL | .7778^*^ | .3557 | .034 | .063 | 1.493 |
|  |  |  |  | WL | .5556 | .3557 | .125 | -.160 | 1.271 |
|  |  |  |  | CK | .8889^*^ | .3557 | .016 | .174 | 1.604 |
|  |  | YL | dimension3 | RL | -.2222 | .3557 | .535 | -.937 | .493 |
|  |  |  |  | BL | -.8889^*^ | .3557 | .016 | -1.604 | -.174 |
|  |  |  |  | GL | -.1111 | .3557 | .756 | -.826 | .604 |
|  |  |  |  | WL | -.3333 | .3557 | .353 | -1.049 | .382 |
|  |  |  |  | CK | .0000 | .3557 | 1.000 | -.715 | .715 |
|  |  | GL | dimension3 | RL | -.1111 | .3557 | .756 | -.826 | .604 |
|  |  |  |  | BL | -.7778^*^ | .3557 | .034 | -1.493 | -.063 |
|  |  |  |  | YL | .1111 | .3557 | .756 | -.604 | .826 |
|  |  |  |  | WL | -.2222 | .3557 | .535 | -.937 | .493 |
|  |  |  |  | CK | .1111 | .3557 | .756 | -.604 | .826 |
|  |  | WL | dimension3 | RL | .1111 | .3557 | .756 | -.604 | .826 |
|  |  |  |  | BL | -.5556 | .3557 | .125 | -1.271 | .160 |
|  |  |  |  | YL | .3333 | .3557 | .353 | -.382 | 1.049 |
|  |  |  |  | GL | .2222 | .3557 | .535 | -.493 | .937 |
|  |  |  |  | CK | .3333 | .3557 | .353 | -.382 | 1.049 |
|  |  | CK | dimension3 | RL | -.2222 | .3557 | .535 | -.937 | .493 |
|  |  |  |  | BL | -.8889^*^ | .3557 | .016 | -1.604 | -.174 |
|  |  |  |  | YL | .0000 | .3557 | 1.000 | -.715 | .715 |
|  |  |  |  | GL | -.1111 | .3557 | .756 | -.826 | .604 |
|  |  |  |  | WL | -.3333 | .3557 | .353 | -1.049 | .382 |

| **Leaf Numbers** | | | | |
| --- | --- | --- | --- | --- |
|  | Light Quality | N | Subset for alpha = 0.05 | |
|  |  |  | 1 | 2 |
| Duncan^a^ | YL | 9 | 5.000 |  |
|  | CK | 9 | 5.000 |  |
|  | GL | 9 | 5.111 |  |
|  | RL | 9 | 5.222 | 5.222 |
|  | WL | 9 | 5.333 | 5.333 |
|  | BL | 9 |  | 5.889 |
|  | Sig. |  | .413 | .082 |

**2. The ANOVA of Leaf Length**

| **ANOVA** | | | | | |
| --- | --- | --- | --- | --- | --- |
| Leaf Length | | | | | |
|  | Sum of squares | df | Mean square | F | Sig. |
| Between Groups | .481 | 5 | .096 | .590 | .708 |
| Within groups | 7.838 | 48 | .163 |  |  |
| Total | 8.319 | 53 |  |  |  |

| **Multiple Comparisons** | | | | | | | | | |
| --- | --- | --- | --- | --- | --- | --- | --- | --- | --- |
| Dependent Variable: Leaf Length | | | | | | | | | |
|  | (I) Light Quality | | (J)  Light Quality | | Mean Difference (I-J) | Std.Errer | Sig. | 95% Confidence Internal | |
|  |  |  |  |  |  |  |  | lower Bound | Upper Bound |
| LSD | dimension2 | RL | dimension3 | BL | -.1000 | .1905 | .602 | -.483 | .283 |
|  |  |  |  | YL | -.1222 | .1905 | .524 | -.505 | .261 |
|  |  |  |  | GL | -.1333 | .1905 | .487 | -.516 | .250 |
|  |  |  |  | WL | .0333 | .1905 | .862 | -.350 | .416 |
|  |  |  |  | CK | -.2556 | .1905 | .186 | -.639 | .127 |
|  |  | BL | dimension3 | RL | .1000 | .1905 | .602 | -.283 | .483 |
|  |  |  |  | YL | -.0222 | .1905 | .908 | -.405 | .361 |
|  |  |  |  | GL | -.0333 | .1905 | .862 | -.416 | .350 |
|  |  |  |  | WL | .1333 | .1905 | .487 | -.250 | .516 |
|  |  |  |  | CK | -.1556 | .1905 | .418 | -.539 | .227 |
|  |  | YL | dimension3 | RL | .1222 | .1905 | .524 | -.261 | .505 |
|  |  |  |  | BL | .0222 | .1905 | .908 | -.361 | .405 |
|  |  |  |  | GL | -.0111 | .1905 | .954 | -.394 | .372 |
|  |  |  |  | WL | .1556 | .1905 | .418 | -.227 | .539 |
|  |  |  |  | CK | -.1333 | .1905 | .487 | -.516 | .250 |
|  |  | GL | dimension3 | RL | .1333 | .1905 | .487 | -.250 | .516 |
|  |  |  |  | BL | .0333 | .1905 | .862 | -.350 | .416 |
|  |  |  |  | YL | .0111 | .1905 | .954 | -.372 | .394 |
|  |  |  |  | WL | .1667 | .1905 | .386 | -.216 | .550 |
|  |  |  |  | CK | -.1222 | .1905 | .524 | -.505 | .261 |
|  |  | WL | dimension3 | RL | -.0333 | .1905 | .862 | -.416 | .350 |
|  |  |  |  | BL | -.1333 | .1905 | .487 | -.516 | .250 |
|  |  |  |  | YL | -.1556 | .1905 | .418 | -.539 | .227 |
|  |  |  |  | GL | -.1667 | .1905 | .386 | -.550 | .216 |
|  |  |  |  | CK | -.2889 | .1905 | .136 | -.672 | .094 |
|  |  | CK | dimension3 | RL | .2556 | .1905 | .186 | -.127 | .639 |
|  |  |  |  | BL | .1556 | .1905 | .418 | -.227 | .539 |
|  |  |  |  | YL | .1333 | .1905 | .487 | -.250 | .516 |
|  |  |  |  | GL | .1222 | .1905 | .524 | -.261 | .505 |
|  |  |  |  | WL | .2889 | .1905 | .136 | -.094 | .672 |

| **Leaf Length** | | | |
| --- | --- | --- | --- |
|  | Light Quality | N | Subset for alpha = 0.05 |
|  |  |  | 1 |
| Duncan^a^ | WL | 9 | 3.167 |
|  | RL | 9 | 3.200 |
|  | BL | 9 | 3.300 |
|  | YL | 9 | 3.322 |
|  | GL | 9 | 3.333 |
|  | CK | 9 | 3.456 |
|  | Sig. |  | .192 |

**3. The ANOVA of Leaf Width**

| **ANOVA** | | | | | |
| --- | --- | --- | --- | --- | --- |
| Leaf Width | | | | | |
|  | Sum of squares | df | Mean square | F | Sig. |
| Between Groups | .430 | 5 | .086 | 1.117 | .364 |
| Within groups | 3.700 | 48 | .077 |  |  |
| Total | 4.130 | 53 |  |  |  |

| **Multiple Comparisons** | | | | | | | | | |
| --- | --- | --- | --- | --- | --- | --- | --- | --- | --- |
| Dependent Variable: Leaf Width | | | | | | | | | |
|  | (I) Light Quality | | (J)  Light Quality | | Mean Difference (I-J) | Std.Errer | Sig. | 95% Confidence Internal | |
|  |  |  |  |  |  |  |  | lower Bound | Upper Bound |
| LSD | dimension2 | RL | dimension3 | BL | -.1556 | .1309 | .240 | -.419 | .108 |
|  |  |  |  | YL | -.0667 | .1309 | .613 | -.330 | .196 |
|  |  |  |  | GL | -.0667 | .1309 | .613 | -.330 | .196 |
|  |  |  |  | WL | .0667 | .1309 | .613 | -.196 | .330 |
|  |  |  |  | CK | -.2000 | .1309 | .133 | -.463 | .063 |
|  |  | BL | dimension3 | RL | .1556 | .1309 | .240 | -.108 | .419 |
|  |  |  |  | YL | .0889 | .1309 | .500 | -.174 | .352 |
|  |  |  |  | GL | .0889 | .1309 | .500 | -.174 | .352 |
|  |  |  |  | WL | .2222 | .1309 | .096 | -.041 | .485 |
|  |  |  |  | CK | -.0444 | .1309 | .736 | -.308 | .219 |
|  |  | YL | dimension3 | RL | .0667 | .1309 | .613 | -.196 | .330 |
|  |  |  |  | BL | -.0889 | .1309 | .500 | -.352 | .174 |
|  |  |  |  | GL | .0000 | .1309 | 1.000 | -.263 | .263 |
|  |  |  |  | WL | .1333 | .1309 | .313 | -.130 | .396 |
|  |  |  |  | CK | -.1333 | .1309 | .313 | -.396 | .130 |
|  |  | GL | dimension3 | RL | .0667 | .1309 | .613 | -.196 | .330 |
|  |  |  |  | BL | -.0889 | .1309 | .500 | -.352 | .174 |
|  |  |  |  | YL | .0000 | .1309 | 1.000 | -.263 | .263 |
|  |  |  |  | WL | .1333 | .1309 | .313 | -.130 | .396 |
|  |  |  |  | CK | -.1333 | .1309 | .313 | -.396 | .130 |
|  |  | WL | dimension3 | RL | -.0667 | .1309 | .613 | -.330 | .196 |
|  |  |  |  | BL | -.2222 | .1309 | .096 | -.485 | .041 |
|  |  |  |  | YL | -.1333 | .1309 | .313 | -.396 | .130 |
|  |  |  |  | GL | -.1333 | .1309 | .313 | -.396 | .130 |
|  |  |  |  | CK | -.2667^*^ | .1309 | .047 | -.530 | -.004 |
|  |  | CK | dimension3 | RL | .2000 | .1309 | .133 | -.063 | .463 |
|  |  |  |  | BL | .0444 | .1309 | .736 | -.219 | .308 |
|  |  |  |  | YL | .1333 | .1309 | .313 | -.130 | .396 |
|  |  |  |  | GL | .1333 | .1309 | .313 | -.130 | .396 |
|  |  |  |  | WL | .2667^*^ | .1309 | .047 | .004 | .530 |

| **Leaf Width** | | | |
| --- | --- | --- | --- |
|  | Light Quality | N | Subset for alpha = 0.05 |
|  |  |  | 1 |
| Duncan^a^ | WL | 9 | 2.122 |
|  | RL | 9 | 2.189 |
|  | GL | 9 | 2.256 |
|  | YL | 9 | 2.256 |
|  | BL | 9 | 2.344 |
|  | CK | 9 | 2.389 |
|  | Sig. |  | .079 |

**4. The ANOVA of Stem Diameter**

| **ANOVA** | | | | | |
| --- | --- | --- | --- | --- | --- |
| Stem Diameter | | | | | |
|  | Sum of squares | df | Mean square | F | Sig. |
| Between Groups | .866 | 5 | .173 | 2.464 | .046 |
| Within groups | 3.373 | 48 | .070 |  |  |
| Total | 4.238 | 53 |  |  |  |

| **Multiple Comparisons** | | | | | | | | | |
| --- | --- | --- | --- | --- | --- | --- | --- | --- | --- |
| Dependent Variable: Stem Diameter | | | | | | | | | |
|  | (I) Light Quality | | (J)  Light Quality | | Mean Difference (I-J) | Std.Errer | Sig. | 95% Confidence Internal | |
|  |  |  |  |  |  |  |  | lower Bound | Upper Bound |
| LSD | dimension2 | RL | dimension3 | BL | -.27222^*^ | .12495 | .034 | -.5235 | -.0210 |
|  |  |  |  | YL | -.05222 | .12495 | .678 | -.3035 | .1990 |
|  |  |  |  | GL | .10000 | .12495 | .427 | -.1512 | .3512 |
|  |  |  |  | WL | .09889 | .12495 | .433 | -.1523 | .3501 |
|  |  |  |  | CK | .03222 | .12495 | .798 | -.2190 | .2835 |
|  |  | BL | dimension3 | RL | .27222^*^ | .12495 | .034 | .0210 | .5235 |
|  |  |  |  | YL | .22000 | .12495 | .085 | -.0312 | .4712 |
|  |  |  |  | GL | .37222^*^ | .12495 | .005 | .1210 | .6235 |
|  |  |  |  | WL | .37111^*^ | .12495 | .005 | .1199 | .6223 |
|  |  |  |  | CK | .30444^*^ | .12495 | .019 | .0532 | .5557 |
|  |  | YL | dimension3 | RL | .05222 | .12495 | .678 | -.1990 | .3035 |
|  |  |  |  | BL | -.22000 | .12495 | .085 | -.4712 | .0312 |
|  |  |  |  | GL | .15222 | .12495 | .229 | -.0990 | .4035 |
|  |  |  |  | WL | .15111 | .12495 | .232 | -.1001 | .4023 |
|  |  |  |  | CK | .08444 | .12495 | .502 | -.1668 | .3357 |
|  |  | GL | dimension3 | RL | -.10000 | .12495 | .427 | -.3512 | .1512 |
|  |  |  |  | BL | -.37222^*^ | .12495 | .005 | -.6235 | -.1210 |
|  |  |  |  | YL | -.15222 | .12495 | .229 | -.4035 | .0990 |
|  |  |  |  | WL | -.00111 | .12495 | .993 | -.2523 | .2501 |
|  |  |  |  | CK | -.06778 | .12495 | .590 | -.3190 | .1835 |
|  |  | WL | dimension3 | RL | -.09889 | .12495 | .433 | -.3501 | .1523 |
|  |  |  |  | BL | -.37111^*^ | .12495 | .005 | -.6223 | -.1199 |
|  |  |  |  | YL | -.15111 | .12495 | .232 | -.4023 | .1001 |
|  |  |  |  | GL | .00111 | .12495 | .993 | -.2501 | .2523 |
|  |  |  |  | CK | -.06667 | .12495 | .596 | -.3179 | .1846 |
|  |  | CK | dimension3 | RL | -.03222 | .12495 | .798 | -.2835 | .2190 |
|  |  |  |  | BL | -.30444^*^ | .12495 | .019 | -.5557 | -.0532 |
|  |  |  |  | YL | -.08444 | .12495 | .502 | -.3357 | .1668 |
|  |  |  |  | GL | .06778 | .12495 | .590 | -.1835 | .3190 |
|  |  |  |  | WL | .06667 | .12495 | .596 | -.1846 | .3179 |

| **Stem Diameter** | | | | |
| --- | --- | --- | --- | --- |
|  | Light Quality | N | Subset for alpha = 0.05 | |
|  |  |  | 1 | 2 |
| Duncan^a^ | GL | 9 | 2.3122 |  |
|  | WL | 9 | 2.3133 |  |
|  | CK | 9 | 2.3800 |  |
|  | RL | 9 | 2.4122 |  |
|  | YL | 9 | 2.4644 | 2.4644 |
|  | BL | 9 |  | 2.6844 |
|  | Sig. |  | .287 | .085 |

**5. The ANOVA of Stem Length**

| **ANOVA** | | | | | |
| --- | --- | --- | --- | --- | --- |
| Stem Length | | | | | |
|  | Sum of squares | df | Mean square | F | Sig. |
| Between Groups | 2.844 | 5 | .569 | .966 | .448 |
| Within groups | 28.267 | 48 | .589 |  |  |
| Total | 31.110 | 53 |  |  |  |

| **Multiple Comparisons** | | | | | | | | | |
| --- | --- | --- | --- | --- | --- | --- | --- | --- | --- |
| Dependent Variable: Stem Length | | | | | | | | | |
|  | (I) Light Quality | | (J)  Light Quality | | Mean Difference (I-J) | Std.Errer | Sig. | 95% Confidence Internal | |
|  |  |  |  |  |  |  |  | lower Bound | Upper Bound |
| LSD | dimension2 | RL | dimension3 | BL | -.6444 | .3618 | .081 | -1.372 | .083 |
|  |  |  |  | YL | -.0778 | .3618 | .831 | -.805 | .650 |
|  |  |  |  | GL | -.1222 | .3618 | .737 | -.850 | .605 |
|  |  |  |  | WL | .0556 | .3618 | .879 | -.672 | .783 |
|  |  |  |  | CK | -.1222 | .3618 | .737 | -.850 | .605 |
|  |  | BL | dimension3 | RL | .6444 | .3618 | .081 | -.083 | 1.372 |
|  |  |  |  | YL | .5667 | .3618 | .124 | -.161 | 1.294 |
|  |  |  |  | GL | .5222 | .3618 | .155 | -.205 | 1.250 |
|  |  |  |  | WL | .7000 | .3618 | .059 | -.027 | 1.427 |
|  |  |  |  | CK | .5222 | .3618 | .155 | -.205 | 1.250 |
|  |  | YL | dimension3 | RL | .0778 | .3618 | .831 | -.650 | .805 |
|  |  |  |  | BL | -.5667 | .3618 | .124 | -1.294 | .161 |
|  |  |  |  | GL | -.0444 | .3618 | .903 | -.772 | .683 |
|  |  |  |  | WL | .1333 | .3618 | .714 | -.594 | .861 |
|  |  |  |  | CK | -.0444 | .3618 | .903 | -.772 | .683 |
|  |  | GL | dimension3 | RL | .1222 | .3618 | .737 | -.605 | .850 |
|  |  |  |  | BL | -.5222 | .3618 | .155 | -1.250 | .205 |
|  |  |  |  | YL | .0444 | .3618 | .903 | -.683 | .772 |
|  |  |  |  | WL | .1778 | .3618 | .625 | -.550 | .905 |
|  |  |  |  | CK | .0000 | .3618 | 1.000 | -.727 | .727 |
|  |  | WL | dimension3 | RL | -.0556 | .3618 | .879 | -.783 | .672 |
|  |  |  |  | BL | -.7000 | .3618 | .059 | -1.427 | .027 |
|  |  |  |  | YL | -.1333 | .3618 | .714 | -.861 | .594 |
|  |  |  |  | GL | -.1778 | .3618 | .625 | -.905 | .550 |
|  |  |  |  | CK | -.1778 | .3618 | .625 | -.905 | .550 |
|  |  | CK | dimension3 | RL | .1222 | .3618 | .737 | -.605 | .850 |
|  |  |  |  | BL | -.5222 | .3618 | .155 | -1.250 | .205 |
|  |  |  |  | YL | .0444 | .3618 | .903 | -.683 | .772 |
|  |  |  |  | GL | .0000 | .3618 | 1.000 | -.727 | .727 |
|  |  |  |  | WL | .1778 | .3618 | .625 | -.550 | .905 |

| **Stem Length** | | | |
| --- | --- | --- | --- |
|  | Light Quality | N | Subset for alpha = 0.05 |
|  |  |  | 1 |
| Duncan^a^ | WL | 9 | 5.833 |
|  | RL | 9 | 5.889 |
|  | YL | 9 | 5.967 |
|  | CK | 9 | 6.011 |
|  | GL | 9 | 6.011 |
|  | BL | 9 | 6.533 |
|  | Sig. |  | .095 |

**6. The ANOVA of Root Numbers**

| **ANOVA** | | | | | |
| --- | --- | --- | --- | --- | --- |
| Root Numbers | | | | | |
|  | Sum of squares | df | Mean square | F | Sig. |
| Between Groups | 5.426 | 5 | 1.085 | 2.321 | .057 |
| Within groups | 22.444 | 48 | .468 |  |  |
| Total | 27.870 | 53 |  |  |  |

| **Multiple Comparisons** | | | | | | | | | |
| --- | --- | --- | --- | --- | --- | --- | --- | --- | --- |
| Dependent Variable: Root Numbers | | | | | | | | | |
|  | (I) Light Quality | | (J)  Light Quality | | Mean Difference (I-J) | Std.Errer | Sig. | 95% Confidence Internal | |
|  |  |  |  |  |  |  |  | lower Bound | Upper Bound |
| LSD | dimension2 | RL | dimension3 | BL | -.1111 | .3223 | .732 | -.759 | .537 |
|  |  |  |  | YL | .5556 | .3223 | .091 | -.093 | 1.204 |
|  |  |  |  | GL | .5556 | .3223 | .091 | -.093 | 1.204 |
|  |  |  |  | WL | -.1111 | .3223 | .732 | -.759 | .537 |
|  |  |  |  | CK | .5556 | .3223 | .091 | -.093 | 1.204 |
|  |  | BL | dimension3 | RL | .1111 | .3223 | .732 | -.537 | .759 |
|  |  |  |  | YL | .6667^*^ | .3223 | .044 | .019 | 1.315 |
|  |  |  |  | GL | .6667^*^ | .3223 | .044 | .019 | 1.315 |
|  |  |  |  | WL | .0000 | .3223 | 1.000 | -.648 | .648 |
|  |  |  |  | CK | .6667^*^ | .3223 | .044 | .019 | 1.315 |
|  |  | YL | dimension3 | RL | -.5556 | .3223 | .091 | -1.204 | .093 |
|  |  |  |  | BL | -.6667^*^ | .3223 | .044 | -1.315 | -.019 |
|  |  |  |  | GL | .0000 | .3223 | 1.000 | -.648 | .648 |
|  |  |  |  | WL | -.6667^*^ | .3223 | .044 | -1.315 | -.019 |
|  |  |  |  | CK | .0000 | .3223 | 1.000 | -.648 | .648 |
|  |  | GL | dimension3 | RL | -.5556 | .3223 | .091 | -1.204 | .093 |
|  |  |  |  | BL | -.6667^*^ | .3223 | .044 | -1.315 | -.019 |
|  |  |  |  | YL | .0000 | .3223 | 1.000 | -.648 | .648 |
|  |  |  |  | WL | -.6667^*^ | .3223 | .044 | -1.315 | -.019 |
|  |  |  |  | CK | .0000 | .3223 | 1.000 | -.648 | .648 |
|  |  | WL | dimension3 | RL | .1111 | .3223 | .732 | -.537 | .759 |
|  |  |  |  | BL | .0000 | .3223 | 1.000 | -.648 | .648 |
|  |  |  |  | YL | .6667^*^ | .3223 | .044 | .019 | 1.315 |
|  |  |  |  | GL | .6667^*^ | .3223 | .044 | .019 | 1.315 |
|  |  |  |  | CK | .6667^*^ | .3223 | .044 | .019 | 1.315 |
|  |  | CK | dimension3 | RL | -.5556 | .3223 | .091 | -1.204 | .093 |
|  |  |  |  | BL | -.6667^*^ | .3223 | .044 | -1.315 | -.019 |
|  |  |  |  | YL | .0000 | .3223 | 1.000 | -.648 | .648 |
|  |  |  |  | GL | .0000 | .3223 | 1.000 | -.648 | .648 |
|  |  |  |  | WL | -.6667^*^ | .3223 | .044 | -1.315 | -.019 |

| **Root Numbers** | | | |
| --- | --- | --- | --- |
|  | Light Quality | N | Subset for alpha = 0.05 |
|  |  |  | 1 |
| Duncan^a^ | YL | 9 | 3.444 |
|  | GL | 9 | 3.444 |
|  | CK | 9 | 3.444 |
|  | RL | 9 | 4.000 |
|  | BL | 9 | 4.111 |
|  | WL | 9 | 4.111 |
|  | Sig. |  | .074 |

**7. The ANOVA of Root Length**

| **ANOVA** | | | | | |
| --- | --- | --- | --- | --- | --- |
| Root Length | | | | | |
|  | Sum of squares | df | Mean square | F | Sig. |
| Between Groups | 9.204 | 5 | 1.841 | 2.372 | .053 |
| Within groups | 37.251 | 48 | .776 |  |  |
| Total | 46.455 | 53 |  |  |  |

| **Multiple Comparisons** | | | | | | | | | |
| --- | --- | --- | --- | --- | --- | --- | --- | --- | --- |
| Dependent Variable: Root Length | | | | | | | | | |
|  | (I) Light Quality | | (J)  Light Quality | | Mean Difference (I-J) | Std.Errer | Sig. | 95% Confidence Internal | |
|  |  |  |  |  |  |  |  | lower Bound | Upper Bound |
| LSD | dimension2 | RL | dimension3 | BL | .1000 | .4153 | .811 | -.735 | .935 |
|  |  |  |  | YL | .0000 | .4153 | 1.000 | -.835 | .835 |
|  |  |  |  | GL | .4778 | .4153 | .256 | -.357 | 1.313 |
|  |  |  |  | WL | .5444 | .4153 | .196 | -.291 | 1.379 |
|  |  |  |  | CK | 1.1667^*^ | .4153 | .007 | .332 | 2.002 |
|  |  | BL | dimension3 | RL | -.1000 | .4153 | .811 | -.935 | .735 |
|  |  |  |  | YL | -.1000 | .4153 | .811 | -.935 | .735 |
|  |  |  |  | GL | .3778 | .4153 | .368 | -.457 | 1.213 |
|  |  |  |  | WL | .4444 | .4153 | .290 | -.391 | 1.279 |
|  |  |  |  | CK | 1.0667^*^ | .4153 | .013 | .232 | 1.902 |
|  |  | YL | dimension3 | RL | .0000 | .4153 | 1.000 | -.835 | .835 |
|  |  |  |  | BL | .1000 | .4153 | .811 | -.735 | .935 |
|  |  |  |  | GL | .4778 | .4153 | .256 | -.357 | 1.313 |
|  |  |  |  | WL | .5444 | .4153 | .196 | -.291 | 1.379 |
|  |  |  |  | CK | 1.1667^*^ | .4153 | .007 | .332 | 2.002 |
|  |  | GL | dimension3 | RL | -.4778 | .4153 | .256 | -1.313 | .357 |
|  |  |  |  | BL | -.3778 | .4153 | .368 | -1.213 | .457 |
|  |  |  |  | YL | -.4778 | .4153 | .256 | -1.313 | .357 |
|  |  |  |  | WL | .0667 | .4153 | .873 | -.768 | .902 |
|  |  |  |  | CK | .6889 | .4153 | .104 | -.146 | 1.524 |
|  |  | WL | dimension3 | RL | -.5444 | .4153 | .196 | -1.379 | .291 |
|  |  |  |  | BL | -.4444 | .4153 | .290 | -1.279 | .391 |
|  |  |  |  | YL | -.5444 | .4153 | .196 | -1.379 | .291 |
|  |  |  |  | GL | -.0667 | .4153 | .873 | -.902 | .768 |
|  |  |  |  | CK | .6222 | .4153 | .141 | -.213 | 1.457 |
|  |  | CK | dimension3 | RL | -1.1667^*^ | .4153 | .007 | -2.002 | -.332 |
|  |  |  |  | BL | -1.0667^*^ | .4153 | .013 | -1.902 | -.232 |
|  |  |  |  | YL | -1.1667^*^ | .4153 | .007 | -2.002 | -.332 |
|  |  |  |  | GL | -.6889 | .4153 | .104 | -1.524 | .146 |
|  |  |  |  | WL | -.6222 | .4153 | .141 | -1.457 | .213 |

| **Root Length** | | | | |
| --- | --- | --- | --- | --- |
|  | Light Quality | N | Subset for alpha = 0.05 | |
|  |  |  | 1 | 2 |
| Duncan^a^ | CK | 9 | 5.367 |  |
|  | WL | 9 | 5.989 | 5.989 |
|  | GL | 9 | 6.056 | 6.056 |
|  | BL | 9 |  | 6.433 |
|  | YL | 9 |  | 6.533 |
|  | RL | 9 |  | 6.533 |
|  | Sig. |  | .123 | .251 |

**8. The ANOVA of Fresh Weight**

| **ANOVA** | | | | | |
| --- | --- | --- | --- | --- | --- |
| Fresh Weight | | | | | |
|  | Sum of squares | df | Mean square | F | Sig. |
| Between Groups | .423 | 5 | .085 | 2.683 | .032 |
| Within groups | 1.515 | 48 | .032 |  |  |
| Total | 1.938 | 53 |  |  |  |

| **Multiple Comparisons** | | | | | | | | | |
| --- | --- | --- | --- | --- | --- | --- | --- | --- | --- |
| Dependent Variable: Fresh Weight | | | | | | | | | |
|  | (I) Light Quality | | (J)  Light Quality | | Mean Difference (I-J) | Std.Errer | Sig. | 95% Confidence Internal | |
|  |  |  |  |  |  |  |  | lower Bound | Upper Bound |
| LSD | dimension2 | RL | dimension3 | BL | -.15111 | .08374 | .077 | -.3195 | .0173 |
|  |  |  |  | YL | -.00667 | .08374 | .937 | -.1750 | .1617 |
|  |  |  |  | GL | .13667 | .08374 | .109 | -.0317 | .3050 |
|  |  |  |  | WL | .02000 | .08374 | .812 | -.1484 | .1884 |
|  |  |  |  | CK | .07778 | .08374 | .358 | -.0906 | .2461 |
|  |  | BL | dimension3 | RL | .15111 | .08374 | .077 | -.0173 | .3195 |
|  |  |  |  | YL | .14444 | .08374 | .091 | -.0239 | .3128 |
|  |  |  |  | GL | .28778^*^ | .08374 | .001 | .1194 | .4561 |
|  |  |  |  | WL | .17111^*^ | .08374 | .047 | .0027 | .3395 |
|  |  |  |  | CK | .22889^*^ | .08374 | .009 | .0605 | .3973 |
|  |  | YL | dimension3 | RL | .00667 | .08374 | .937 | -.1617 | .1750 |
|  |  |  |  | BL | -.14444 | .08374 | .091 | -.3128 | .0239 |
|  |  |  |  | GL | .14333 | .08374 | .093 | -.0250 | .3117 |
|  |  |  |  | WL | .02667 | .08374 | .752 | -.1417 | .1950 |
|  |  |  |  | CK | .08444 | .08374 | .318 | -.0839 | .2528 |
|  |  | GL | dimension3 | RL | -.13667 | .08374 | .109 | -.3050 | .0317 |
|  |  |  |  | BL | -.28778^*^ | .08374 | .001 | -.4561 | -.1194 |
|  |  |  |  | YL | -.14333 | .08374 | .093 | -.3117 | .0250 |
|  |  |  |  | WL | -.11667 | .08374 | .170 | -.2850 | .0517 |
|  |  |  |  | CK | -.05889 | .08374 | .485 | -.2273 | .1095 |
|  |  | WL | dimension3 | RL | -.02000 | .08374 | .812 | -.1884 | .1484 |
|  |  |  |  | BL | -.17111^*^ | .08374 | .047 | -.3395 | -.0027 |
|  |  |  |  | YL | -.02667 | .08374 | .752 | -.1950 | .1417 |
|  |  |  |  | GL | .11667 | .08374 | .170 | -.0517 | .2850 |
|  |  |  |  | CK | .05778 | .08374 | .494 | -.1106 | .2261 |
|  |  | CK | dimension3 | RL | -.07778 | .08374 | .358 | -.2461 | .0906 |
|  |  |  |  | BL | -.22889^*^ | .08374 | .009 | -.3973 | -.0605 |
|  |  |  |  | YL | -.08444 | .08374 | .318 | -.2528 | .0839 |
|  |  |  |  | GL | .05889 | .08374 | .485 | -.1095 | .2273 |
|  |  |  |  | WL | -.05778 | .08374 | .494 | -.2261 | .1106 |

| **Fresh Weight** | | | | |
| --- | --- | --- | --- | --- |
|  | Light Quality | N | Subset for alpha = 0.05 | |
|  |  |  | 1 | 2 |
| Duncan^a^ | GL | 9 | 1.2889 |  |
|  | CK | 9 | 1.3478 |  |
|  | WL | 9 | 1.4056 | 1.4056 |
|  | RL | 9 | 1.4256 | 1.4256 |
|  | YL | 9 | 1.4322 | 1.4322 |
|  | BL | 9 |  | 1.5767 |
|  | Sig. |  | .134 | .067 |

**9. The ANOVA of Dry Weight**

| **ANOVA** | | | | | |
| --- | --- | --- | --- | --- | --- |
| Dry Weight | | | | | |
|  | Sum of squares | df | Mean square | F | Sig. |
| Between Groups | .016 | 5 | .003 | 4.196 | .003 |
| Within groups | .037 | 48 | .001 |  |  |
| Total | .054 | 53 |  |  |  |

| **Multiple Comparisons** | | | | | | | | | |
| --- | --- | --- | --- | --- | --- | --- | --- | --- | --- |
| Dependent Variable: Dry Weight | | | | | | | | | |
|  | (I) Light Quality | | (J)  Light Quality | | Mean Difference (I-J) | Std.Errer | Sig. | 95% Confidence Internal | |
|  |  |  |  |  |  |  |  | lower Bound | Upper Bound |
| LSD | dimension2 | RL | dimension3 | BL | -.038889^*^ | .013139 | .005 | -.06531 | -.01247 |
|  |  |  |  | YL | -.020000 | .013139 | .135 | -.04642 | .00642 |
|  |  |  |  | GL | .008889 | .013139 | .502 | -.01753 | .03531 |
|  |  |  |  | WL | .003333 | .013139 | .801 | -.02308 | .02975 |
|  |  |  |  | CK | .007778 | .013139 | .557 | -.01864 | .03420 |
|  |  | BL | dimension3 | RL | .038889^*^ | .013139 | .005 | .01247 | .06531 |
|  |  |  |  | YL | .018889 | .013139 | .157 | -.00753 | .04531 |
|  |  |  |  | GL | .047778^*^ | .013139 | .001 | .02136 | .07420 |
|  |  |  |  | WL | .042222^*^ | .013139 | .002 | .01580 | .06864 |
|  |  |  |  | CK | .046667^*^ | .013139 | .001 | .02025 | .07308 |
|  |  | YL | dimension3 | RL | .020000 | .013139 | .135 | -.00642 | .04642 |
|  |  |  |  | BL | -.018889 | .013139 | .157 | -.04531 | .00753 |
|  |  |  |  | GL | .028889^*^ | .013139 | .033 | .00247 | .05531 |
|  |  |  |  | WL | .023333 | .013139 | .082 | -.00308 | .04975 |
|  |  |  |  | CK | .027778^*^ | .013139 | .040 | .00136 | .05420 |
|  |  | GL | dimension3 | RL | -.008889 | .013139 | .502 | -.03531 | .01753 |
|  |  |  |  | BL | -.047778^*^ | .013139 | .001 | -.07420 | -.02136 |
|  |  |  |  | YL | -.028889^*^ | .013139 | .033 | -.05531 | -.00247 |
|  |  |  |  | WL | -.005556 | .013139 | .674 | -.03197 | .02086 |
|  |  |  |  | CK | -.001111 | .013139 | .933 | -.02753 | .02531 |
|  |  | WL | dimension3 | RL | -.003333 | .013139 | .801 | -.02975 | .02308 |
|  |  |  |  | BL | -.042222^*^ | .013139 | .002 | -.06864 | -.01580 |
|  |  |  |  | YL | -.023333 | .013139 | .082 | -.04975 | .00308 |
|  |  |  |  | GL | .005556 | .013139 | .674 | -.02086 | .03197 |
|  |  |  |  | CK | .004444 | .013139 | .737 | -.02197 | .03086 |
|  |  | CK | dimension3 | RL | -.007778 | .013139 | .557 | -.03420 | .01864 |
|  |  |  |  | BL | -.046667^*^ | .013139 | .001 | -.07308 | -.02025 |
|  |  |  |  | YL | -.027778^*^ | .013139 | .040 | -.05420 | -.00136 |
|  |  |  |  | GL | .001111 | .013139 | .933 | -.02531 | .02753 |
|  |  |  |  | WL | -.004444 | .013139 | .737 | -.03086 | .02197 |

| **Dry Weight** | | | | |
| --- | --- | --- | --- | --- |
|  | Light Quality | N | Subset for alpha = 0.05 | |
|  |  |  | 1 | 2 |
| Duncan^a^ | GL | 9 | .15222 |  |
|  | CK | 9 | .15333 |  |
|  | WL | 9 | .15778 |  |
|  | RL | 9 | .16111 |  |
|  | YL | 9 | .18111 | .18111 |
|  | BL | 9 |  | .20000 |
|  | Sig. |  | .054 | .157 |

**10. The ANOVA of Chlorophyll a**

| **ANOVA** | | | | | |
| --- | --- | --- | --- | --- | --- |
| Chlorophyll a | | | | | |
|  | Sum of squares | df | Mean square | F | Sig. |
| Between Groups | .227 | 5 | .045 | 4.274 | .018 |
| Within groups | .128 | 12 | .011 |  |  |
| Total | .355 | 17 |  |  |  |

| **Multiple Comparisons** | | | | | | | | | |
| --- | --- | --- | --- | --- | --- | --- | --- | --- | --- |
| Dependent Variable: Chlorophyll a | | | | | | | | | |
|  | (I) Light Quality | | (J)  Light Quality | | Mean Difference (I-J) | Std.Errer | Sig. | 95% Confidence Internal | |
|  |  |  |  |  |  |  |  | lower Bound | Upper Bound |
| LSD | dimension2 | RL | dimension3 | BL | -.31177^*^ | .08422 | .003 | -.4953 | -.1283 |
|  |  |  |  | YL | -.14523 | .08422 | .110 | -.3287 | .0383 |
|  |  |  |  | GL | -.28128^*^ | .08422 | .006 | -.4648 | -.0978 |
|  |  |  |  | WL | -.26020^*^ | .08422 | .009 | -.4437 | -.0767 |
|  |  |  |  | CK | -.08981 | .08422 | .307 | -.2733 | .0937 |
|  |  | BL | dimension3 | RL | .31177^*^ | .08422 | .003 | .1283 | .4953 |
|  |  |  |  | YL | .16654 | .08422 | .071 | -.0169 | .3500 |
|  |  |  |  | GL | .03050 | .08422 | .724 | -.1530 | .2140 |
|  |  |  |  | WL | .05157 | .08422 | .552 | -.1319 | .2351 |
|  |  |  |  | CK | .22196^*^ | .08422 | .022 | .0385 | .4055 |
|  |  | YL | dimension3 | RL | .14523 | .08422 | .110 | -.0383 | .3287 |
|  |  |  |  | BL | -.16654 | .08422 | .071 | -.3500 | .0169 |
|  |  |  |  | GL | -.13605 | .08422 | .132 | -.3195 | .0474 |
|  |  |  |  | WL | -.11497 | .08422 | .197 | -.2985 | .0685 |
|  |  |  |  | CK | .05542 | .08422 | .523 | -.1281 | .2389 |
|  |  | GL | dimension3 | RL | .28128^*^ | .08422 | .006 | .0978 | .4648 |
|  |  |  |  | BL | -.03050 | .08422 | .724 | -.2140 | .1530 |
|  |  |  |  | YL | .13605 | .08422 | .132 | -.0474 | .3195 |
|  |  |  |  | WL | .02108 | .08422 | .807 | -.1624 | .2046 |
|  |  |  |  | CK | .19147^*^ | .08422 | .042 | .0080 | .3750 |
|  |  | WL | dimension3 | RL | .26020^*^ | .08422 | .009 | .0767 | .4437 |
|  |  |  |  | BL | -.05157 | .08422 | .552 | -.2351 | .1319 |
|  |  |  |  | YL | .11497 | .08422 | .197 | -.0685 | .2985 |
|  |  |  |  | GL | -.02108 | .08422 | .807 | -.2046 | .1624 |
|  |  |  |  | CK | .17039 | .08422 | .066 | -.0131 | .3539 |
|  |  | CK | dimension3 | RL | .08981 | .08422 | .307 | -.0937 | .2733 |
|  |  |  |  | BL | -.22196^*^ | .08422 | .022 | -.4055 | -.0385 |
|  |  |  |  | YL | -.05542 | .08422 | .523 | -.2389 | .1281 |
|  |  |  |  | GL | -.19147^*^ | .08422 | .042 | -.3750 | -.0080 |
|  |  |  |  | WL | -.17039 | .08422 | .066 | -.3539 | .0131 |

| **Chlorophyll a** | | | | | |
| --- | --- | --- | --- | --- | --- |
|  | Light Quality | N | Subset for alpha = 0.05 | | |
|  |  |  | 1 | 2 | 3 |
| Duncan^a^ | RL | 3 | .5035 |  |  |
|  | CK | 3 | .5933 | .5933 |  |
|  | YL | 3 | .6487 | .6487 | .6487 |
|  | WL | 3 |  | .7637 | .7637 |
|  | GL | 3 |  | .7848 | .7848 |
|  | BL | 3 |  |  | .8153 |
|  | Sig. |  | .126 | .056 | .091 |

**11. The ANOVA of Chlorophyll b**

| **ANOVA** | | | | | |
| --- | --- | --- | --- | --- | --- |
| Chlorophyll b | | | | | |
|  | Sum of squares | df | Mean square | F | Sig. |
| Between Groups | .024 | 5 | .005 | 1.381 | .298 |
| Within groups | .041 | 12 | .003 |  |  |
| Total | .065 | 17 |  |  |  |

| **Multiple Comparisons** | | | | | | | | | |
| --- | --- | --- | --- | --- | --- | --- | --- | --- | --- |
| Dependent Variable: Chlorophyll b | | | | | | | | | |
|  | (I) Light Quality | | (J)  Light Quality | | Mean Difference (I-J) | Std.Errer | Sig. | 95% Confidence Internal | |
|  |  |  |  |  |  |  |  | lower Bound | Upper Bound |
| LSD | dimension2 | RL | dimension3 | BL | -.08282 | .04793 | .110 | -.1872 | .0216 |
|  |  |  |  | YL | -.03588 | .04793 | .469 | -.1403 | .0685 |
|  |  |  |  | GL | -.10327 | .04793 | .052 | -.2077 | .0012 |
|  |  |  |  | WL | -.08052 | .04793 | .119 | -.1849 | .0239 |
|  |  |  |  | CK | -.09404 | .04793 | .073 | -.1985 | .0104 |
|  |  | BL | dimension3 | RL | .08282 | .04793 | .110 | -.0216 | .1872 |
|  |  |  |  | YL | .04694 | .04793 | .347 | -.0575 | .1514 |
|  |  |  |  | GL | -.02045 | .04793 | .677 | -.1249 | .0840 |
|  |  |  |  | WL | .00229 | .04793 | .963 | -.1021 | .1067 |
|  |  |  |  | CK | -.01123 | .04793 | .819 | -.1157 | .0932 |
|  |  | YL | dimension3 | RL | .03588 | .04793 | .469 | -.0685 | .1403 |
|  |  |  |  | BL | -.04694 | .04793 | .347 | -.1514 | .0575 |
|  |  |  |  | GL | -.06739 | .04793 | .185 | -.1718 | .0370 |
|  |  |  |  | WL | -.04464 | .04793 | .370 | -.1491 | .0598 |
|  |  |  |  | CK | -.05816 | .04793 | .248 | -.1626 | .0463 |
|  |  | GL | dimension3 | RL | .10327 | .04793 | .052 | -.0012 | .2077 |
|  |  |  |  | BL | .02045 | .04793 | .677 | -.0840 | .1249 |
|  |  |  |  | YL | .06739 | .04793 | .185 | -.0370 | .1718 |
|  |  |  |  | WL | .02275 | .04793 | .644 | -.0817 | .1272 |
|  |  |  |  | CK | .00922 | .04793 | .851 | -.0952 | .1137 |
|  |  | WL | dimension3 | RL | .08052 | .04793 | .119 | -.0239 | .1849 |
|  |  |  |  | BL | -.00229 | .04793 | .963 | -.1067 | .1021 |
|  |  |  |  | YL | .04464 | .04793 | .370 | -.0598 | .1491 |
|  |  |  |  | GL | -.02275 | .04793 | .644 | -.1272 | .0817 |
|  |  |  |  | CK | -.01352 | .04793 | .783 | -.1179 | .0909 |
|  |  | CK | dimension3 | RL | .09404 | .04793 | .073 | -.0104 | .1985 |
|  |  |  |  | BL | .01123 | .04793 | .819 | -.0932 | .1157 |
|  |  |  |  | YL | .05816 | .04793 | .248 | -.0463 | .1626 |
|  |  |  |  | GL | -.00922 | .04793 | .851 | -.1137 | .0952 |
|  |  |  |  | WL | .01352 | .04793 | .783 | -.0909 | .1179 |

| **Chlorophyll b** | | | |
| --- | --- | --- | --- |
|  | Light Quality | N | Subset for alpha = 0.05 |
|  |  |  | 1 |
| Duncan^a^ | RL | 3 | .2873 |
|  | YL | 3 | .3232 |
|  | WL | 3 | .3679 |
|  | BL | 3 | .3702 |
|  | CK | 3 | .3814 |
|  | GL | 3 | .3906 |
|  | Sig. |  | .075 |

**12. The ANOVA of Chlorophyll a+b**

| **ANOVA** | | | | | |
| --- | --- | --- | --- | --- | --- |
| Chlorophyll a+b | | | | | |
|  | Sum of squares | df | Mean square | F | Sig. |
| Between Groups | .356 | 5 | .071 | 2.801 | .067 |
| Within groups | .305 | 12 | .025 |  |  |
| Total | .662 | 17 |  |  |  |

| **Multiple Comparisons** | | | | | | | | | |
| --- | --- | --- | --- | --- | --- | --- | --- | --- | --- |
| Dependent Variable: Chlorophyll a+b | | | | | | | | | |
|  | (I) Light Quality | | (J)  Light Quality | | Mean Difference (I-J) | Std.Errer | Sig. | 95% Confidence Internal | |
|  |  |  |  |  |  |  |  | lower Bound | Upper Bound |
| LSD | dimension2 | RL | dimension3 | BL | -.394590^*^ | .130257 | .010 | -.67840 | -.11078 |
|  |  |  |  | YL | -.181110 | .130257 | .190 | -.46492 | .10270 |
|  |  |  |  | GL | -.384544^*^ | .130257 | .012 | -.66835 | -.10074 |
|  |  |  |  | WL | -.340723^*^ | .130257 | .023 | -.62453 | -.05692 |
|  |  |  |  | CK | -.183850 | .130257 | .184 | -.46766 | .09996 |
|  |  | BL | dimension3 | RL | .394590^*^ | .130257 | .010 | .11078 | .67840 |
|  |  |  |  | YL | .213480 | .130257 | .127 | -.07033 | .49729 |
|  |  |  |  | GL | .010046 | .130257 | .940 | -.27376 | .29385 |
|  |  |  |  | WL | .053866 | .130257 | .687 | -.22994 | .33767 |
|  |  |  |  | CK | .210740 | .130257 | .132 | -.07307 | .49455 |
|  |  | YL | dimension3 | RL | .181110 | .130257 | .190 | -.10270 | .46492 |
|  |  |  |  | BL | -.213480 | .130257 | .127 | -.49729 | .07033 |
|  |  |  |  | GL | -.203434 | .130257 | .144 | -.48724 | .08037 |
|  |  |  |  | WL | -.159613 | .130257 | .244 | -.44342 | .12419 |
|  |  |  |  | CK | -.002740 | .130257 | .984 | -.28655 | .28107 |
|  |  | GL | dimension3 | RL | .384544^*^ | .130257 | .012 | .10074 | .66835 |
|  |  |  |  | BL | -.010046 | .130257 | .940 | -.29385 | .27376 |
|  |  |  |  | YL | .203434 | .130257 | .144 | -.08037 | .48724 |
|  |  |  |  | WL | .043820 | .130257 | .742 | -.23999 | .32763 |
|  |  |  |  | CK | .200694 | .130257 | .149 | -.08311 | .48450 |
|  |  | WL | dimension3 | RL | .340723^*^ | .130257 | .023 | .05692 | .62453 |
|  |  |  |  | BL | -.053866 | .130257 | .687 | -.33767 | .22994 |
|  |  |  |  | YL | .159613 | .130257 | .244 | -.12419 | .44342 |
|  |  |  |  | GL | -.043820 | .130257 | .742 | -.32763 | .23999 |
|  |  |  |  | CK | .156873 | .130257 | .252 | -.12693 | .44068 |
|  |  | CK | dimension3 | RL | .183850 | .130257 | .184 | -.09996 | .46766 |
|  |  |  |  | BL | -.210740 | .130257 | .132 | -.49455 | .07307 |
|  |  |  |  | YL | .002740 | .130257 | .984 | -.28107 | .28655 |
|  |  |  |  | GL | -.200694 | .130257 | .149 | -.48450 | .08311 |
|  |  |  |  | WL | -.156873 | .130257 | .252 | -.44068 | .12693 |

| **Chlorophyll a+b** | | | | |
| --- | --- | --- | --- | --- |
|  | Light Quality | N | Subset for alpha = 0.05 | |
|  |  |  | 1 | 2 |
| Duncan^a^ | RL | 3 | .79083 |  |
|  | YL | 3 | .97194 | .97194 |
|  | CK | 3 | .97468 | .97468 |
|  | WL | 3 |  | 1.13155 |
|  | GL | 3 |  | 1.17538 |
|  | BL | 3 |  | 1.18542 |
|  | Sig. |  | .204 | .160 |

**13. The ANOVA of Yield**

| **ANOVA** | | | | | |
| --- | --- | --- | --- | --- | --- |
| Yield | | | | | |
|  | Sum of squares | df | Mean square | F | Sig. |
| Between Groups | .435 | 5 | .087 | 7.645 | .000 |
| Within groups | .341 | 30 | .011 |  |  |
| Total | .776 | 35 |  |  |  |

| **Multiple Comparisons** | | | | | | | | | |
| --- | --- | --- | --- | --- | --- | --- | --- | --- | --- |
| Dependent Variable:Yield | | | | | | | | | |
|  | (I) Light Quality | | (J)  Light Quality | | Mean Difference (I-J) | Std.Errer | Sig. | 95% Confidence Internal | |
|  |  |  |  |  |  |  |  | lower Bound | Upper Bound |
| LSD | dimension2 | RL | dimension3 | BL | -.23250^*^ | .06159 | .001 | -.3583 | -.1067 |
|  |  |  |  | YL | -.29583^*^ | .06159 | .000 | -.4216 | -.1700 |
|  |  |  |  | GL | -.05433 | .06159 | .385 | -.1801 | .0715 |
|  |  |  |  | WL | -.02067 | .06159 | .740 | -.1465 | .1051 |
|  |  |  |  | CK | -.14133^*^ | .06159 | .029 | -.2671 | -.0155 |
|  |  | BL | dimension3 | RL | .23250^*^ | .06159 | .001 | .1067 | .3583 |
|  |  |  |  | YL | -.06333 | .06159 | .312 | -.1891 | .0625 |
|  |  |  |  | GL | .17817^*^ | .06159 | .007 | .0524 | .3040 |
|  |  |  |  | WL | .21183^*^ | .06159 | .002 | .0860 | .3376 |
|  |  |  |  | CK | .09117 | .06159 | .149 | -.0346 | .2170 |
|  |  | YL | dimension3 | RL | .29583^*^ | .06159 | .000 | .1700 | .4216 |
|  |  |  |  | BL | .06333 | .06159 | .312 | -.0625 | .1891 |
|  |  |  |  | GL | .24150^*^ | .06159 | .000 | .1157 | .3673 |
|  |  |  |  | WL | .27517^*^ | .06159 | .000 | .1494 | .4010 |
|  |  |  |  | CK | .15450^*^ | .06159 | .018 | .0287 | .2803 |
|  |  | GL | dimension3 | RL | .05433 | .06159 | .385 | -.0715 | .1801 |
|  |  |  |  | BL | -.17817^*^ | .06159 | .007 | -.3040 | -.0524 |
|  |  |  |  | YL | -.24150^*^ | .06159 | .000 | -.3673 | -.1157 |
|  |  |  |  | WL | .03367 | .06159 | .589 | -.0921 | .1595 |
|  |  |  |  | CK | -.08700 | .06159 | .168 | -.2128 | .0388 |
|  |  | WL | dimension3 | RL | .02067 | .06159 | .740 | -.1051 | .1465 |
|  |  |  |  | BL | -.21183^*^ | .06159 | .002 | -.3376 | -.0860 |
|  |  |  |  | YL | -.27517^*^ | .06159 | .000 | -.4010 | -.1494 |
|  |  |  |  | GL | -.03367 | .06159 | .589 | -.1595 | .0921 |
|  |  |  |  | CK | -.12067 | .06159 | .059 | -.2465 | .0051 |
|  |  | CK | dimension3 | RL | .14133^*^ | .06159 | .029 | .0155 | .2671 |
|  |  |  |  | BL | -.09117 | .06159 | .149 | -.2170 | .0346 |
|  |  |  |  | YL | -.15450^*^ | .06159 | .018 | -.2803 | -.0287 |
|  |  |  |  | GL | .08700 | .06159 | .168 | -.0388 | .2128 |
|  |  |  |  | WL | .12067 | .06159 | .059 | -.0051 | .2465 |

| **Yield** | | | | | | |
| --- | --- | --- | --- | --- | --- | --- |
|  | Light Quality | N | Subset for alpha = 0.05 | | | |
|  |  |  | 1 | 2 | 3 | 4 |
| Duncan^a^ | RL | 6 | .3240 |  |  |  |
|  | WL | 6 | .3447 | .3447 |  |  |
|  | GL | 6 | .3783 | .3783 |  |  |
|  | CK | 6 |  | .4653 | .4653 |  |
|  | BL | 6 |  |  | .5565 | .5565 |
|  | YL | 6 |  |  |  | .6198 |
|  | Sig. |  | .413 | .073 | .149 | .312 |

**14. The ANOVA of qP**

| **ANOVA** | | | | | |
| --- | --- | --- | --- | --- | --- |
| qP | | | | | |
|  | Sum of squares | df | Mean square | F | Sig. |
| Between Groups | .248 | 5 | .050 | .776 | .575 |
| Within groups | 1.921 | 30 | .064 |  |  |
| Total | 2.169 | 35 |  |  |  |

| **Multiple Comparisons** | | | | | | | | | |
| --- | --- | --- | --- | --- | --- | --- | --- | --- | --- |
| Dependent Variable:qP | | | | | | | | | |
|  | (I) Light Quality | | (J)  Light Quality | | Mean Difference (I-J) | Std.Errer | Sig. | 95% Confidence Internal | |
|  |  |  |  |  |  |  |  | lower Bound | Upper Bound |
| LSD | dimension2 | RL | dimension3 | BL | -.20183 | .14608 | .177 | -.5002 | .0965 |
|  |  |  |  | YL | -.15350 | .14608 | .302 | -.4518 | .1448 |
|  |  |  |  | GL | .01200 | .14608 | .935 | -.2863 | .3103 |
|  |  |  |  | WL | -.11033 | .14608 | .456 | -.4087 | .1880 |
|  |  |  |  | CK | -.00617 | .14608 | .967 | -.3045 | .2922 |
|  |  | BL | dimension3 | RL | .20183 | .14608 | .177 | -.0965 | .5002 |
|  |  |  |  | YL | .04833 | .14608 | .743 | -.2500 | .3467 |
|  |  |  |  | GL | .21383 | .14608 | .154 | -.0845 | .5122 |
|  |  |  |  | WL | .09150 | .14608 | .536 | -.2068 | .3898 |
|  |  |  |  | CK | .19567 | .14608 | .190 | -.1027 | .4940 |
|  |  | YL | dimension3 | RL | .15350 | .14608 | .302 | -.1448 | .4518 |
|  |  |  |  | BL | -.04833 | .14608 | .743 | -.3467 | .2500 |
|  |  |  |  | GL | .16550 | .14608 | .266 | -.1328 | .4638 |
|  |  |  |  | WL | .04317 | .14608 | .770 | -.2552 | .3415 |
|  |  |  |  | CK | .14733 | .14608 | .321 | -.1510 | .4457 |
|  |  | GL | dimension3 | RL | -.01200 | .14608 | .935 | -.3103 | .2863 |
|  |  |  |  | BL | -.21383 | .14608 | .154 | -.5122 | .0845 |
|  |  |  |  | YL | -.16550 | .14608 | .266 | -.4638 | .1328 |
|  |  |  |  | WL | -.12233 | .14608 | .409 | -.4207 | .1760 |
|  |  |  |  | CK | -.01817 | .14608 | .902 | -.3165 | .2802 |
|  |  | WL | dimension3 | RL | .11033 | .14608 | .456 | -.1880 | .4087 |
|  |  |  |  | BL | -.09150 | .14608 | .536 | -.3898 | .2068 |
|  |  |  |  | YL | -.04317 | .14608 | .770 | -.3415 | .2552 |
|  |  |  |  | GL | .12233 | .14608 | .409 | -.1760 | .4207 |
|  |  |  |  | CK | .10417 | .14608 | .481 | -.1942 | .4025 |
|  |  | CK | dimension3 | RL | .00617 | .14608 | .967 | -.2922 | .3045 |
|  |  |  |  | BL | -.19567 | .14608 | .190 | -.4940 | .1027 |
|  |  |  |  | YL | -.14733 | .14608 | .321 | -.4457 | .1510 |
|  |  |  |  | GL | .01817 | .14608 | .902 | -.2802 | .3165 |
|  |  |  |  | WL | -.10417 | .14608 | .481 | -.4025 | .1942 |

| **qP** | | | |
| --- | --- | --- | --- |
|  | Light Quality | N | Subset for alpha = 0.05 |
|  |  |  | 1 |
| Duncan^a^ | GL | 6 | .6735 |
|  | RL | 6 | .6855 |
|  | CK | 6 | .6917 |
|  | WL | 6 | .7958 |
|  | YL | 6 | .8390 |
|  | BL | 6 | .8873 |
|  | Sig. |  | .208 |

**15. The ANOVA of qN**

| **ANOVA** | | | | | |
| --- | --- | --- | --- | --- | --- |
| qN | | | | | |
|  | Sum of squares | df | Mean square | F | Sig. |
| Between Groups | .999 | 5 | .200 | 6.068 | .001 |
| Within groups | .988 | 30 | .033 |  |  |
| Total | 1.986 | 35 |  |  |  |

| **Multiple Comparisons** | | | | | | | | | |
| --- | --- | --- | --- | --- | --- | --- | --- | --- | --- |
| Dependent Variable:qN | | | | | | | | | |
|  | (I) Light Quality | | (J)  Light Quality | | Mean Difference (I-J) | Std.Errer | Sig. | 95% Confidence Internal | |
|  |  |  |  |  |  |  |  | lower Bound | Upper Bound |
| LSD | dimension2 | RL | dimension3 | BL | .13583 | .10475 | .205 | -.0781 | .3498 |
|  |  |  |  | YL | .37100^*^ | .10475 | .001 | .1571 | .5849 |
|  |  |  |  | GL | .00867 | .10475 | .935 | -.2053 | .2226 |
|  |  |  |  | WL | -.12800 | .10475 | .231 | -.3419 | .0859 |
|  |  |  |  | CK | .24700^*^ | .10475 | .025 | .0331 | .4609 |
|  |  | BL | dimension3 | RL | -.13583 | .10475 | .205 | -.3498 | .0781 |
|  |  |  |  | YL | .23517^*^ | .10475 | .032 | .0212 | .4491 |
|  |  |  |  | GL | -.12717 | .10475 | .234 | -.3411 | .0868 |
|  |  |  |  | WL | -.26383^*^ | .10475 | .017 | -.4778 | -.0499 |
|  |  |  |  | CK | .11117 | .10475 | .297 | -.1028 | .3251 |
|  |  | YL | dimension3 | RL | -.37100^*^ | .10475 | .001 | -.5849 | -.1571 |
|  |  |  |  | BL | -.23517^*^ | .10475 | .032 | -.4491 | -.0212 |
|  |  |  |  | GL | -.36233^*^ | .10475 | .002 | -.5763 | -.1484 |
|  |  |  |  | WL | -.49900^*^ | .10475 | .000 | -.7129 | -.2851 |
|  |  |  |  | CK | -.12400 | .10475 | .246 | -.3379 | .0899 |
|  |  | GL | dimension3 | RL | -.00867 | .10475 | .935 | -.2226 | .2053 |
|  |  |  |  | BL | .12717 | .10475 | .234 | -.0868 | .3411 |
|  |  |  |  | YL | .36233^*^ | .10475 | .002 | .1484 | .5763 |
|  |  |  |  | WL | -.13667 | .10475 | .202 | -.3506 | .0773 |
|  |  |  |  | CK | .23833^*^ | .10475 | .030 | .0244 | .4523 |
|  |  | WL | dimension3 | RL | .12800 | .10475 | .231 | -.0859 | .3419 |
|  |  |  |  | BL | .26383^*^ | .10475 | .017 | .0499 | .4778 |
|  |  |  |  | YL | .49900^*^ | .10475 | .000 | .2851 | .7129 |
|  |  |  |  | GL | .13667 | .10475 | .202 | -.0773 | .3506 |
|  |  |  |  | CK | .37500^*^ | .10475 | .001 | .1611 | .5889 |
|  |  | CK | dimension3 | RL | -.24700^*^ | .10475 | .025 | -.4609 | -.0331 |
|  |  |  |  | BL | -.11117 | .10475 | .297 | -.3251 | .1028 |
|  |  |  |  | YL | .12400 | .10475 | .246 | -.0899 | .3379 |
|  |  |  |  | GL | -.23833^*^ | .10475 | .030 | -.4523 | -.0244 |
|  |  |  |  | WL | -.37500^*^ | .10475 | .001 | -.5889 | -.1611 |

| **qN** | | | | | | |
| --- | --- | --- | --- | --- | --- | --- |
|  | Light Quality | N | Subset for alpha = 0.05 | | | |
|  |  |  | 1 | 2 | 3 | 4 |
| Duncan^a^ | YL | 6 | .3187 |  |  |  |
|  | CK | 6 | .4427 | .4427 |  |  |
|  | BL | 6 |  | .5538 | .5538 |  |
|  | GL | 6 |  |  | .6810 | .6810 |
|  | RL | 6 |  |  | .6897 | .6897 |
|  | WL | 6 |  |  |  | .8177 |
|  | Sig. |  | .246 | .297 | .231 | .228 |

**16. The ANOVA of Soluble Sugar**

| **ANOVA** | | | | | |
| --- | --- | --- | --- | --- | --- |
| Soluble Sugar | | | | | |
|  | Sum of squares | df | Mean square | F | Sig. |
| Between Groups | 249.392 | 5 | 49.878 | 7.373 | .002 |
| Within groups | 81.181 | 12 | 6.765 |  |  |
| Total | 330.574 | 17 |  |  |  |

| **Multiple Comparisons** | | | | | | | | | | | | | | |
| --- | --- | --- | --- | --- | --- | --- | --- | --- | --- | --- | --- | --- | --- | --- |
| Dependent Variable: Soluble Sugar | | | | | | | | | | | | | | |
|  | (I) Light Quality | | (J)  Light Quality | | Mean Difference (I-J) | Std.Errer | | Sig. | | 95% Confidence Internal | | | |  |
|  |  |  |  |  |  |  |  |  |  | lower Bound | | Upper Bound | |  |
| LSD | dimension2 | RL | dimension3 | BL | 3.13500 | | 2.12369 | | .166 | | -1.4921 | | 7.7621 | |
|  |  |  |  | YL | -1.99167 | | 2.12369 | | .367 | | -6.6188 | | 2.6355 | |
|  |  |  |  | GL | 2.42167 | | 2.12369 | | .276 | | -2.2055 | | 7.0488 | |
|  |  |  |  | WL | 9.99000^*^ | | 2.12369 | | .001 | | 5.3629 | | 14.6171 | |
|  |  |  |  | CK | 3.42000 | | 2.12369 | | .133 | | -1.2071 | | 8.0471 | |
|  |  | BL | dimension3 | RL | -3.13500 | | 2.12369 | | .166 | | -7.7621 | | 1.4921 | |
|  |  |  |  | YL | -5.12667^*^ | | 2.12369 | | .033 | | -9.7538 | | -.4995 | |
|  |  |  |  | GL | -.71333 | | 2.12369 | | .743 | | -5.3405 | | 3.9138 | |
|  |  |  |  | WL | 6.85500^*^ | | 2.12369 | | .007 | | 2.2279 | | 11.4821 | |
|  |  |  |  | CK | .28500 | | 2.12369 | | .895 | | -4.3421 | | 4.9121 | |
|  |  | YL | dimension3 | RL | 1.99167 | | 2.12369 | | .367 | | -2.6355 | | 6.6188 | |
|  |  |  |  | BL | 5.12667^*^ | | 2.12369 | | .033 | | .4995 | | 9.7538 | |
|  |  |  |  | GL | 4.41333 | | 2.12369 | | .060 | | -.2138 | | 9.0405 | |
|  |  |  |  | WL | 11.98167^*^ | | 2.12369 | | .000 | | 7.3545 | | 16.6088 | |
|  |  |  |  | CK | 5.41167^*^ | | 2.12369 | | .026 | | .7845 | | 10.0388 | |
|  |  | GL | dimension3 | RL | -2.42167 | | 2.12369 | | .276 | | -7.0488 | | 2.2055 | |
|  |  |  |  | BL | .71333 | | 2.12369 | | .743 | | -3.9138 | | 5.3405 | |
|  |  |  |  | YL | -4.41333 | | 2.12369 | | .060 | | -9.0405 | | .2138 | |
|  |  |  |  | WL | 7.56833^*^ | | 2.12369 | | .004 | | 2.9412 | | 12.1955 | |
|  |  |  |  | CK | .99833 | | 2.12369 | | .647 | | -3.6288 | | 5.6255 | |
|  |  | WL | dimension3 | RL | -9.99000^*^ | | 2.12369 | | .001 | | -14.6171 | | -5.3629 | |
|  |  |  |  | BL | -6.85500^*^ | | 2.12369 | | .007 | | -11.4821 | | -2.2279 | |
|  |  |  |  | YL | -11.98167^*^ | | 2.12369 | | .000 | | -16.6088 | | -7.3545 | |
|  |  |  |  | GL | -7.56833^*^ | | 2.12369 | | .004 | | -12.1955 | | -2.9412 | |
|  |  |  |  | CK | -6.57000^*^ | | 2.12369 | | .009 | | -11.1971 | | -1.9429 | |
|  |  | CK | dimension3 | RL | -3.42000 | | 2.12369 | | .133 | | -8.0471 | | 1.2071 | |
|  |  |  |  | BL | -.28500 | | 2.12369 | | .895 | | -4.9121 | | 4.3421 | |
|  |  |  |  | YL | -5.41167^*^ | | 2.12369 | | .026 | | -10.0388 | | -.7845 | |
|  |  |  |  | GL | -.99833 | | 2.12369 | | .647 | | -5.6255 | | 3.6288 | |
|  |  |  |  | WL | 6.57000^*^ | | 2.12369 | | .009 | | 1.9429 | | 11.1971 | |

| **Soluble Sugar** | | | | | |
| --- | --- | --- | --- | --- | --- |
|  | Light Quality | N | Subset for alpha = 0.05 | | |
|  |  |  | 1 | 2 | 3 |
| Duncan^a^ | WL | 3 | 20.5267 |  |  |
|  | CK | 3 |  | 27.0967 |  |
|  | BL | 3 |  | 27.3817 |  |
|  | GL | 3 |  | 28.0950 | 28.0950 |
|  | RL | 3 |  | 30.5167 | 30.5167 |
|  | YL | 3 |  |  | 32.5083 |
|  | Sig. |  | 1.000 | .161 | .071 |

**17. The ANOVA of Reducing Sugar**

| **ANOVA** | | | | | |
| --- | --- | --- | --- | --- | --- |
| Reducing Sugar | | | | | |
|  | Sum of squares | df | Mean square | F | Sig. |
| Between Groups | 4.349 | 5 | .870 | 10.924 | .000 |
| Within groups | .955 | 12 | .080 |  |  |
| Total | 5.305 | 17 |  |  |  |

| **Multiple Comparisons** | | | | | | | | | |
| --- | --- | --- | --- | --- | --- | --- | --- | --- | --- |
| Dependent Variable: Reducing Sugar | | | | | | | | | |
|  | (I) Light Quality | | (J)  Light Quality | | Mean Difference (I-J) | Std.Errer | Sig. | 95% Confidence Internal | |
|  |  |  |  |  |  |  |  | lower Bound | Upper Bound |
| LSD | dimension2 | RL | dimension3 | BL | .44333 | .23040 | .078 | -.2604 | 1.1471 |
|  |  |  |  | YL | .24278 | .23040 | .313 | -.4610 | .9465 |
|  |  |  |  | GL | .58167 | .23040 | .027 | -.1221 | 1.2854 |
|  |  |  |  | WL | 1.47333^*^ | .23040 | .000 | .7696 | 2.1771 |
|  |  |  |  | CK | .07333 | .23040 | .756 | -.6304 | .7771 |
|  |  | BL | dimension3 | RL | -.44333 | .23040 | .078 | -1.1471 | .2604 |
|  |  |  |  | YL | -.20056 | .23040 | .401 | -.9043 | .5032 |
|  |  |  |  | GL | .13833 | .23040 | .559 | -.5654 | .8421 |
|  |  |  |  | WL | 1.03000^*^ | .23040 | .001 | .3262 | 1.7338 |
|  |  |  |  | CK | -.37000 | .23040 | .134 | -1.0738 | .3338 |
|  |  | YL | dimension3 | RL | -.24278 | .23040 | .313 | -.9465 | .4610 |
|  |  |  |  | BL | .20056 | .23040 | .401 | -.5032 | .9043 |
|  |  |  |  | GL | .33889 | .23040 | .167 | -.3649 | 1.0426 |
|  |  |  |  | WL | 1.23056^*^ | .23040 | .000 | .5268 | 1.9343 |
|  |  |  |  | CK | -.16944 | .23040 | .476 | -.8732 | .5343 |
|  |  | GL | dimension3 | RL | -.58167 | .23040 | .027 | -1.2854 | .1221 |
|  |  |  |  | BL | -.13833 | .23040 | .559 | -.8421 | .5654 |
|  |  |  |  | YL | -.33889 | .23040 | .167 | -1.0426 | .3649 |
|  |  |  |  | WL | .89167^*^ | .23040 | .002 | .1879 | 1.5954 |
|  |  |  |  | CK | -.50833 | .23040 | .048 | -1.2121 | .1954 |
|  |  | WL | dimension3 | RL | -1.47333^*^ | .23040 | .000 | -2.1771 | -.7696 |
|  |  |  |  | BL | -1.03000^*^ | .23040 | .001 | -1.7338 | -.3262 |
|  |  |  |  | YL | -1.23056^*^ | .23040 | .000 | -1.9343 | -.5268 |
|  |  |  |  | GL | -.89167^*^ | .23040 | .002 | -1.5954 | -.1879 |
|  |  |  |  | CK | -1.40000^*^ | .23040 | .000 | -2.1038 | -.6962 |
|  |  | CK | dimension3 | RL | -.07333 | .23040 | .756 | -.7771 | .6304 |
|  |  |  |  | BL | .37000 | .23040 | .134 | -.3338 | 1.0738 |
|  |  |  |  | YL | .16944 | .23040 | .476 | -.5343 | .8732 |
|  |  |  |  | GL | .50833 | .23040 | .048 | -.1954 | 1.2121 |
|  |  |  |  | WL | 1.40000^*^ | .23040 | .000 | .6962 | 2.1038 |

| **Reducing Sugar** | | | | |
| --- | --- | --- | --- | --- |
|  | Light Quality | N | alpha = 0.01 的子集 | |
|  |  |  | 1 | 2 |
| Duncan^a^ | WL | 3 | 4.1439 |  |
|  | GL | 3 |  | 5.0356 |
|  | BL | 3 |  | 5.1739 |
|  | YL | 3 |  | 5.3744 |
|  | CK | 3 |  | 5.5439 |
|  | RL | 3 |  | 5.6172 |
|  | Sig. |  | 1.000 | .040 |

**18. The ANOVA of Polysaccharide**

| **ANOVA** | | | | | |
| --- | --- | --- | --- | --- | --- |
| Polysaccharide | | | | | |
|  | Sum of squares | df | Mean square | F | Sig. |
| Between Groups | 197.821 | 5 | 39.564 | 5.861 | .006 |
| Within groups | 81.009 | 12 | 6.751 |  |  |
| Total | 278.830 | 17 |  |  |  |

| **Multiple Comparisons** | | | | | | | | | | | | | | |
| --- | --- | --- | --- | --- | --- | --- | --- | --- | --- | --- | --- | --- | --- | --- |
| Dependent Variable: Polysaccharide | | | | | | | | | | | | | | |
|  | (I) Light Quality | | (J)  Light Quality | | Mean Difference (I-J) | Std.Errer | | Sig. | | 95% Confidence Internal | | | |  |
|  |  |  |  |  |  |  |  |  |  | lower Bound | | Upper Bound | |  |
| LSD | dimension2 | RL | dimension3 | BL | 2.69167 | | 2.12144 | | .229 | | -1.9306 | | 7.3139 | |
|  |  |  |  | YL | -2.23444 | | 2.12144 | | .313 | | -6.8567 | | 2.3878 | |
|  |  |  |  | GL | 1.84000 | | 2.12144 | | .403 | | -2.7822 | | 6.4622 | |
|  |  |  |  | WL | 8.51667^*^ | | 2.12144 | | .002 | | 3.8944 | | 13.1389 | |
|  |  |  |  | CK | 3.34833 | | 2.12144 | | .140 | | -1.2739 | | 7.9706 | |
|  |  | BL | dimension3 | RL | -2.69167 | | 2.12144 | | .229 | | -7.3139 | | 1.9306 | |
|  |  |  |  | YL | -4.92611^*^ | | 2.12144 | | .039 | | -9.5483 | | -.3039 | |
|  |  |  |  | GL | -.85167 | | 2.12144 | | .695 | | -5.4739 | | 3.7706 | |
|  |  |  |  | WL | 5.82500^*^ | | 2.12144 | | .018 | | 1.2028 | | 10.4472 | |
|  |  |  |  | CK | .65667 | | 2.12144 | | .762 | | -3.9656 | | 5.2789 | |
|  |  | YL | dimension3 | RL | 2.23444 | | 2.12144 | | .313 | | -2.3878 | | 6.8567 | |
|  |  |  |  | BL | 4.92611^*^ | | 2.12144 | | .039 | | .3039 | | 9.5483 | |
|  |  |  |  | GL | 4.07444 | | 2.12144 | | .079 | | -.5478 | | 8.6967 | |
|  |  |  |  | WL | 10.75111^*^ | | 2.12144 | | .000 | | 6.1289 | | 15.3733 | |
|  |  |  |  | CK | 5.58278^*^ | | 2.12144 | | .022 | | .9605 | | 10.2050 | |
|  |  | GL | dimension3 | RL | -1.84000 | | 2.12144 | | .403 | | -6.4622 | | 2.7822 | |
|  |  |  |  | BL | .85167 | | 2.12144 | | .695 | | -3.7706 | | 5.4739 | |
|  |  |  |  | YL | -4.07444 | | 2.12144 | | .079 | | -8.6967 | | .5478 | |
|  |  |  |  | WL | 6.67667^*^ | | 2.12144 | | .008 | | 2.0544 | | 11.2989 | |
|  |  |  |  | CK | 1.50833 | | 2.12144 | | .491 | | -3.1139 | | 6.1306 | |
|  |  | WL | dimension3 | RL | -8.51667^*^ | | 2.12144 | | .002 | | -13.1389 | | -3.8944 | |
|  |  |  |  | BL | -5.82500^*^ | | 2.12144 | | .018 | | -10.4472 | | -1.2028 | |
|  |  |  |  | YL | -10.75111^*^ | | 2.12144 | | .000 | | -15.3733 | | -6.1289 | |
|  |  |  |  | GL | -6.67667^*^ | | 2.12144 | | .008 | | -11.2989 | | -2.0544 | |
|  |  |  |  | CK | -5.16833^*^ | | 2.12144 | | .031 | | -9.7906 | | -.5461 | |
|  |  | CK | dimension3 | RL | -3.34833 | | 2.12144 | | .140 | | -7.9706 | | 1.2739 | |
|  |  |  |  | BL | -.65667 | | 2.12144 | | .762 | | -5.2789 | | 3.9656 | |
|  |  |  |  | YL | -5.58278^*^ | | 2.12144 | | .022 | | -10.2050 | | -.9605 | |
|  |  |  |  | GL | -1.50833 | | 2.12144 | | .491 | | -6.1306 | | 3.1139 | |
|  |  |  |  | WL | 5.16833^*^ | | 2.12144 | | .031 | | .5461 | | 9.7906 | |

| **Polysaccharide** | | | | | |
| --- | --- | --- | --- | --- | --- |
|  | Light Quality | N | Subset for alpha = 0.05 | | |
|  |  |  | 1 | 2 | 3 |
| Duncan^a^ | WL | 3 | 16.3828 |  |  |
|  | CK | 3 |  | 21.5511 |  |
|  | BL | 3 |  | 22.2078 | 22.2078 |
|  | GL | 3 |  | 23.0594 | 23.0594 |
|  | RL | 3 |  | 24.8994 | 24.8994 |
|  | YL | 3 |  |  | 27.1339 |
|  | Sig. |  | 1.000 | .169 | .052 |

**19. The ANOVA of Stomatal Area**

| **ANOVA** | | | | | |
| --- | --- | --- | --- | --- | --- |
| Stomatal Area | | | | | |
|  | Sum of squares | df | Mean square | F | Sig. |
| Between Groups | 262449.432 | 5 | 52489.886 | 2.242 | .063 |
| Within groups | 1264265.652 | 54 | 23412.327 |  |  |
| Total | 1526715.084 | 59 |  |  |  |

| **Multiple Comparisons** | | | | | | | | | | | | | |  |
| --- | --- | --- | --- | --- | --- | --- | --- | --- | --- | --- | --- | --- | --- | --- |
| Dependent Variable: Stomatal Area | | | | | | | | | | | | | |  |
|  | (I) Light Quality | | (J)  Light Quality | | Mean Difference (I-J) | | Std.Errer | Sig. | | 95% Confidence Internal | | | | |
|  |  |  |  |  |  |  |  |  |  | lower Bound | | Upper Bound | | |
| LSD | dimension2 | RL | dimension3 | BL | 74.289590 | 68.428542 | | | .282 | | -62.90138 | | 211.48056 |  |
|  |  |  |  | YL | 196.266310^*^ | 68.428542 | | | .006 | | 59.07534 | | 333.45728 |  |
|  |  |  |  | GL | 144.752660^*^ | 68.428542 | | | .039 | | 7.56169 | | 281.94363 |  |
|  |  |  |  | WL | 29.927430 | 68.428542 | | | .664 | | -107.26354 | | 167.11840 |  |
|  |  |  |  | CK | 91.116770 | 68.428542 | | | .189 | | -46.07420 | | 228.30774 |  |
|  |  | BL | dimension3 | RL | -74.289590 | 68.428542 | | | .282 | | -211.48056 | | 62.90138 |  |
|  |  |  |  | YL | 121.976720 | 68.428542 | | | .080 | | -15.21425 | | 259.16769 |  |
|  |  |  |  | GL | 70.463070 | 68.428542 | | | .308 | | -66.72790 | | 207.65404 |  |
|  |  |  |  | WL | -44.362160 | 68.428542 | | | .520 | | -181.55313 | | 92.82881 |  |
|  |  |  |  | CK | 16.827180 | 68.428542 | | | .807 | | -120.36379 | | 154.01815 |  |
|  |  | YL | dimension3 | RL | -196.266310^*^ | 68.428542 | | | .006 | | -333.45728 | | -59.07534 |  |
|  |  |  |  | BL | -121.976720 | 68.428542 | | | .080 | | -259.16769 | | 15.21425 |  |
|  |  |  |  | GL | -51.513650 | 68.428542 | | | .455 | | -188.70462 | | 85.67732 |  |
|  |  |  |  | WL | -166.338880^*^ | 68.428542 | | | .018 | | -303.52985 | | -29.14791 |  |
|  |  |  |  | CK | -105.149540 | 68.428542 | | | .130 | | -242.34051 | | 32.04143 |  |
|  |  | GL | dimension3 | RL | -144.752660^*^ | 68.428542 | | | .039 | | -281.94363 | | -7.56169 |  |
|  |  |  |  | BL | -70.463070 | 68.428542 | | | .308 | | -207.65404 | | 66.72790 |  |
|  |  |  |  | YL | 51.513650 | 68.428542 | | | .455 | | -85.67732 | | 188.70462 |  |
|  |  |  |  | WL | -114.825230 | 68.428542 | | | .099 | | -252.01620 | | 22.36574 |  |
|  |  |  |  | CK | -53.635890 | 68.428542 | | | .437 | | -190.82686 | | 83.55508 |  |
|  |  | WL | dimension3 | RL | -29.927430 | 68.428542 | | | .664 | | -167.11840 | | 107.26354 |  |
|  |  |  |  | BL | 44.362160 | 68.428542 | | | .520 | | -92.82881 | | 181.55313 |  |
|  |  |  |  | YL | 166.338880^*^ | 68.428542 | | | .018 | | 29.14791 | | 303.52985 |  |
|  |  |  |  | GL | 114.825230 | 68.428542 | | | .099 | | -22.36574 | | 252.01620 |  |
|  |  |  |  | CK | 61.189340 | 68.428542 | | | .375 | | -76.00163 | | 198.38031 |  |
|  |  | CK | dimension3 | RL | -91.116770 | 68.428542 | | | .189 | | -228.30774 | | 46.07420 |  |
|  |  |  |  | BL | -16.827180 | 68.428542 | | | .807 | | -154.01815 | | 120.36379 |  |
|  |  |  |  | YL | 105.149540 | 68.428542 | | | .130 | | -32.04143 | | 242.34051 |  |
|  |  |  |  | GL | 53.635890 | 68.428542 | | | .437 | | -83.55508 | | 190.82686 |  |
|  |  |  |  | WL | -61.189340 | 68.428542 | | | .375 | | -198.38031 | | 76.00163 |  |

| **Stomatal Area** | | | | |
| --- | --- | --- | --- | --- |
|  | Light Quality | N | Subset for alpha = 0.05 | |
|  |  |  | 1 | 2 |
| Duncan^a^ | YL | 10 | 1173.25339 |  |
|  | GL | 10 | 1224.76704 | 1224.76704 |
|  | CK | 10 | 1278.40293 | 1278.40293 |
|  | BL | 10 | 1295.23011 | 1295.23011 |
|  | WL | 10 |  | 1339.59227 |
|  | RL | 10 |  | 1369.51970 |
|  | Sig. |  | .109 | .063 |

**20. The ANOVA of Stomatal Density**

| **ANOVA** | | | | | |
| --- | --- | --- | --- | --- | --- |
| Stomatal Density | | | | | |
|  | Sum of squares | df | Mean square | F | Sig. |
| Between Groups | 60.733 | 5 | 12.147 | 5.350 | .000 |
| Within groups | 122.600 | 54 | 2.270 |  |  |
| Total | 183.333 | 59 |  |  |  |

| **Multiple Comparisons** | | | | | | | | | | | | | |  |
| --- | --- | --- | --- | --- | --- | --- | --- | --- | --- | --- | --- | --- | --- | --- |
| Dependent Variable: Stomatal Density | | | | | | | | | | | | | |  |
|  | (I) Light Quality | | (J)  Light Quality | | Mean Difference (I-J) | Std.Errer | | Sig. | | 95% Confidence Internal | | | | |
|  |  |  |  |  |  |  |  |  |  | lower Bound | | Upper Bound | | |
| LSD | dimension2 | RL | dimension3 | BL | -1.300000 | | .673850 | | .059 | | -2.65099 | | .05099 |  |
|  |  |  |  | YL | -2.700000^*^ | | .673850 | | .000 | | -4.05099 | | -1.34901 |  |
|  |  |  |  | GL | -1.100000 | | .673850 | | .108 | | -2.45099 | | .25099 |  |
|  |  |  |  | WL | .200000 | | .673850 | | .768 | | -1.15099 | | 1.55099 |  |
|  |  |  |  | CK | -.100000 | | .673850 | | .883 | | -1.45099 | | 1.25099 |  |
|  |  | BL | dimension3 | RL | 1.300000 | | .673850 | | .059 | | -.05099 | | 2.65099 |  |
|  |  |  |  | YL | -1.400000^*^ | | .673850 | | .043 | | -2.75099 | | -.04901 |  |
|  |  |  |  | GL | .200000 | | .673850 | | .768 | | -1.15099 | | 1.55099 |  |
|  |  |  |  | WL | 1.500000^*^ | | .673850 | | .030 | | .14901 | | 2.85099 |  |
|  |  |  |  | CK | 1.200000 | | .673850 | | .081 | | -.15099 | | 2.55099 |  |
|  |  | YL | dimension3 | RL | 2.700000^*^ | | .673850 | | .000 | | 1.34901 | | 4.05099 |  |
|  |  |  |  | BL | 1.400000^*^ | | .673850 | | .043 | | .04901 | | 2.75099 |  |
|  |  |  |  | GL | 1.600000^*^ | | .673850 | | .021 | | .24901 | | 2.95099 |  |
|  |  |  |  | WL | 2.900000^*^ | | .673850 | | .000 | | 1.54901 | | 4.25099 |  |
|  |  |  |  | CK | 2.600000^*^ | | .673850 | | .000 | | 1.24901 | | 3.95099 |  |
|  |  | GL | dimension3 | RL | 1.100000 | | .673850 | | .108 | | -.25099 | | 2.45099 |  |
|  |  |  |  | BL | -.200000 | | .673850 | | .768 | | -1.55099 | | 1.15099 |  |
|  |  |  |  | YL | -1.600000^*^ | | .673850 | | .021 | | -2.95099 | | -.24901 |  |
|  |  |  |  | WL | 1.300000 | | .673850 | | .059 | | -.05099 | | 2.65099 |  |
|  |  |  |  | CK | 1.000000 | | .673850 | | .144 | | -.35099 | | 2.35099 |  |
|  |  | WL | dimension3 | RL | -.200000 | | .673850 | | .768 | | -1.55099 | | 1.15099 |  |
|  |  |  |  | BL | -1.500000^*^ | | .673850 | | .030 | | -2.85099 | | -.14901 |  |
|  |  |  |  | YL | -2.900000^*^ | | .673850 | | .000 | | -4.25099 | | -1.54901 |  |
|  |  |  |  | GL | -1.300000 | | .673850 | | .059 | | -2.65099 | | .05099 |  |
|  |  |  |  | CK | -.300000 | | .673850 | | .658 | | -1.65099 | | 1.05099 |  |
|  |  | CK | dimension3 | RL | .100000 | | .673850 | | .883 | | -1.25099 | | 1.45099 |  |
|  |  |  |  | BL | -1.200000 | | .673850 | | .081 | | -2.55099 | | .15099 |  |
|  |  |  |  | YL | -2.600000^*^ | | .673850 | | .000 | | -3.95099 | | -1.24901 |  |
|  |  |  |  | GL | -1.000000 | | .673850 | | .144 | | -2.35099 | | .35099 |  |
|  |  |  |  | WL | .300000 | | .673850 | | .658 | | -1.05099 | | 1.65099 |  |

| **Stomatal Density** | | | | |
| --- | --- | --- | --- | --- |
|  | Light Quality | N | Subset for alpha = 0.05 | |
|  |  |  | 1 | 2 |
| Duncan^a^ | WL | 10 | 8.30000 |  |
|  | RL | 10 | 8.50000 |  |
|  | CK | 10 | 8.60000 |  |
|  | GL | 10 | 9.60000 |  |
|  | BL | 10 | 9.80000 |  |
|  | YL | 10 |  | 11.20000 |
|  | Sig. |  | .050 | 1.000 |

**21. The ANOVA of Root Vitality**

| **ANOVA** | | | | | |
| --- | --- | --- | --- | --- | --- |
| Root Vitality | | | | | |
|  | Sum of squares | df | Mean square | F | Sig. |
| Between Groups | .081 | 5 | .016 | 4.793 | .012 |
| Within groups | .041 | 12 | .003 |  |  |
| Total | .122 | 17 |  |  |  |

| **Multiple Comparisons** | | | | | | | | | |
| --- | --- | --- | --- | --- | --- | --- | --- | --- | --- |
| Dependent Variable: Root Vitality | | | | | | | | | |
|  | (I) Light Quality | | (J)  Light Quality | | Mean Difference (I-J) | Std.Errer | Sig. | 95% Confidence Internal | |
|  |  |  |  |  |  |  |  | lower Bound | Upper Bound |
| LSD | dimension2 | RL | dimension3 | BL | -.02162 | .04754 | .657 | -.1252 | .0820 |
|  |  |  |  | YL | -.20155^*^ | .04754 | .001 | -.3051 | -.0980 |
|  |  |  |  | GL | -.13198^*^ | .04754 | .017 | -.2356 | -.0284 |
|  |  |  |  | WL | -.07845 | .04754 | .125 | -.1820 | .0251 |
|  |  |  |  | CK | -.08215 | .04754 | .110 | -.1857 | .0214 |
|  |  | BL | dimension3 | RL | .02162 | .04754 | .657 | -.0820 | .1252 |
|  |  |  |  | YL | -.17993^*^ | .04754 | .003 | -.2835 | -.0764 |
|  |  |  |  | GL | -.11037^*^ | .04754 | .039 | -.2139 | -.0068 |
|  |  |  |  | WL | -.05683 | .04754 | .255 | -.1604 | .0467 |
|  |  |  |  | CK | -.06053 | .04754 | .227 | -.1641 | .0430 |
|  |  | YL | dimension3 | RL | .20155^*^ | .04754 | .001 | .0980 | .3051 |
|  |  |  |  | BL | .17993^*^ | .04754 | .003 | .0764 | .2835 |
|  |  |  |  | GL | .06957 | .04754 | .169 | -.0340 | .1731 |
|  |  |  |  | WL | .12310^*^ | .04754 | .024 | .0195 | .2267 |
|  |  |  |  | CK | .11940^*^ | .04754 | .027 | .0158 | .2230 |
|  |  | GL | dimension3 | RL | .13198^*^ | .04754 | .017 | .0284 | .2356 |
|  |  |  |  | BL | .11037^*^ | .04754 | .039 | .0068 | .2139 |
|  |  |  |  | YL | -.06957 | .04754 | .169 | -.1731 | .0340 |
|  |  |  |  | WL | .05353 | .04754 | .282 | -.0500 | .1571 |
|  |  |  |  | CK | .04983 | .04754 | .315 | -.0537 | .1534 |
|  |  | WL | dimension3 | RL | .07845 | .04754 | .125 | -.0251 | .1820 |
|  |  |  |  | BL | .05683 | .04754 | .255 | -.0467 | .1604 |
|  |  |  |  | YL | -.12310^*^ | .04754 | .024 | -.2267 | -.0195 |
|  |  |  |  | GL | -.05353 | .04754 | .282 | -.1571 | .0500 |
|  |  |  |  | CK | -.00370 | .04754 | .939 | -.1073 | .0999 |
|  |  | CK | dimension3 | RL | .08215 | .04754 | .110 | -.0214 | .1857 |
|  |  |  |  | BL | .06053 | .04754 | .227 | -.0430 | .1641 |
|  |  |  |  | YL | -.11940^*^ | .04754 | .027 | -.2230 | -.0158 |
|  |  |  |  | GL | -.04983 | .04754 | .315 | -.1534 | .0537 |
|  |  |  |  | WL | .00370 | .04754 | .939 | -.0999 | .1073 |

| **Root Vitality** | | | | | |
| --- | --- | --- | --- | --- | --- |
|  | Light Quality | N | Subset for alpha = 0.05 | | |
|  |  |  | 1 | 2 | 3 |
| Duncan^a^ | RL | 3 | .3289 |  |  |
|  | BL | 3 | .3505 | .3505 |  |
|  | WL | 3 | .4073 | .4073 |  |
|  | CK | 3 | .4110 | .4110 |  |
|  | GL | 3 |  | .4608 | .4608 |
|  | YL | 3 |  |  | .5304 |
|  | Sig. |  | .135 | .052 | .169 |

**22. The ANOVA of Soluble Protein**

| **ANOVA** | | | | | |
| --- | --- | --- | --- | --- | --- |
| Soluble Protein | | | | | |
|  | Sum of squares | df | Mean square | F | Sig. |
| Between Groups | 1.364 | 5 | .273 | 3.780 | .027 |
| Within groups | .866 | 12 | .072 |  |  |
| Total | 2.230 | 17 |  |  |  |

| **Multiple Comparisons** | | | | | | | | | |
| --- | --- | --- | --- | --- | --- | --- | --- | --- | --- |
| Dependent Variable: Soluble Protein | | | | | | | | | |
|  | (I) Light Quality | | (J)  Light Quality | | Mean Difference (I-J) | Std.Errer | Sig. | 95% Confidence Internal | |
|  |  |  |  |  |  |  |  | lower Bound | Upper Bound |
| LSD | dimension2 | RL | dimension3 | BL | .53333^*^ | .21936 | .032 | .0554 | 1.0113 |
|  |  |  |  | YL | .20000 | .21936 | .380 | -.2779 | .6779 |
|  |  |  |  | GL | -.30000 | .21936 | .196 | -.7779 | .1779 |
|  |  |  |  | WL | .11667 | .21936 | .605 | -.3613 | .5946 |
|  |  |  |  | CK | -.21333 | .21936 | .350 | -.6913 | .2646 |
|  |  | BL | dimension3 | RL | -.53333^*^ | .21936 | .032 | -1.0113 | -.0554 |
|  |  |  |  | YL | -.33333 | .21936 | .155 | -.8113 | .1446 |
|  |  |  |  | GL | -.83333^*^ | .21936 | .003 | -1.3113 | -.3554 |
|  |  |  |  | WL | -.41667 | .21936 | .082 | -.8946 | .0613 |
|  |  |  |  | CK | -.74667^*^ | .21936 | .005 | -1.2246 | -.2687 |
|  |  | YL | dimension3 | RL | -.20000 | .21936 | .380 | -.6779 | .2779 |
|  |  |  |  | BL | .33333 | .21936 | .155 | -.1446 | .8113 |
|  |  |  |  | GL | -.50000^*^ | .21936 | .042 | -.9779 | -.0221 |
|  |  |  |  | WL | -.08333 | .21936 | .711 | -.5613 | .3946 |
|  |  |  |  | CK | -.41333 | .21936 | .084 | -.8913 | .0646 |
|  |  | GL | dimension3 | RL | .30000 | .21936 | .196 | -.1779 | .7779 |
|  |  |  |  | BL | .83333^*^ | .21936 | .003 | .3554 | 1.3113 |
|  |  |  |  | YL | .50000^*^ | .21936 | .042 | .0221 | .9779 |
|  |  |  |  | WL | .41667 | .21936 | .082 | -.0613 | .8946 |
|  |  |  |  | CK | .08667 | .21936 | .700 | -.3913 | .5646 |
|  |  | WL | dimension3 | RL | -.11667 | .21936 | .605 | -.5946 | .3613 |
|  |  |  |  | BL | .41667 | .21936 | .082 | -.0613 | .8946 |
|  |  |  |  | YL | .08333 | .21936 | .711 | -.3946 | .5613 |
|  |  |  |  | GL | -.41667 | .21936 | .082 | -.8946 | .0613 |
|  |  |  |  | CK | -.33000 | .21936 | .158 | -.8079 | .1479 |
|  |  | CK | dimension3 | RL | .21333 | .21936 | .350 | -.2646 | .6913 |
|  |  |  |  | BL | .74667^*^ | .21936 | .005 | .2687 | 1.2246 |
|  |  |  |  | YL | .41333 | .21936 | .084 | -.0646 | .8913 |
|  |  |  |  | GL | -.08667 | .21936 | .700 | -.5646 | .3913 |
|  |  |  |  | WL | .33000 | .21936 | .158 | -.1479 | .8079 |

| **Soluble Protein** | | | | |
| --- | --- | --- | --- | --- |
|  | Light Quality | N | Subset for alpha = 0.05 | |
|  |  |  | 1 | 2 |
| Duncan^a^ | BL | 3 | 2.3000 |  |
|  | YL | 3 | 2.6333 | 2.6333 |
|  | WL | 3 | 2.7167 | 2.7167 |
|  | RL | 3 |  | 2.8333 |
|  | CK | 3 |  | 3.0467 |
|  | GL | 3 |  | 3.1333 |
|  | Sig. |  | .095 | .059 |

**23. The ANOVA of Total Flavonoid**

| **ANOVA** | | | | | |
| --- | --- | --- | --- | --- | --- |
| Total Flavonoid | | | | | |
|  | Sum of squares | df | Mean square | F | Sig. |
| Between Groups | .342 | 5 | .068 | 5.196 | .009 |
| Within groups | .158 | 12 | .013 |  |  |
| Total | .499 | 17 |  |  |  |

| **Multiple Comparisons** | | | | | | | | | |
| --- | --- | --- | --- | --- | --- | --- | --- | --- | --- |
| Dependent Variable: Total Flavonoid | | | | | | | | | |
|  | (I) Light Quality | | (J)  Light Quality | | Mean Difference (I-J) | Std.Errer | Sig. | 95% Confidence Internal | |
|  |  |  |  |  |  |  |  | lower Bound | Upper Bound |
| LSD | dimension2 | RL | dimension3 | BL | -.21333^*^ | .09361 | .042 | -.4173 | -.0094 |
|  |  |  |  | YL | .15333 | .09361 | .127 | -.0506 | .3573 |
|  |  |  |  | GL | .02333 | .09361 | .807 | -.1806 | .2273 |
|  |  |  |  | WL | .18333 | .09361 | .074 | -.0206 | .3873 |
|  |  |  |  | CK | .16333 | .09361 | .107 | -.0406 | .3673 |
|  |  | BL | dimension3 | RL | .21333^*^ | .09361 | .042 | .0094 | .4173 |
|  |  |  |  | YL | .36667^*^ | .09361 | .002 | .1627 | .5706 |
|  |  |  |  | GL | .23667^*^ | .09361 | .027 | .0327 | .4406 |
|  |  |  |  | WL | .39667^*^ | .09361 | .001 | .1927 | .6006 |
|  |  |  |  | CK | .37667^*^ | .09361 | .002 | .1727 | .5806 |
|  |  | YL | dimension3 | RL | -.15333 | .09361 | .127 | -.3573 | .0506 |
|  |  |  |  | BL | -.36667^*^ | .09361 | .002 | -.5706 | -.1627 |
|  |  |  |  | GL | -.13000 | .09361 | .190 | -.3340 | .0740 |
|  |  |  |  | WL | .03000 | .09361 | .754 | -.1740 | .2340 |
|  |  |  |  | CK | .01000 | .09361 | .917 | -.1940 | .2140 |
|  |  | GL | dimension3 | RL | -.02333 | .09361 | .807 | -.2273 | .1806 |
|  |  |  |  | BL | -.23667^*^ | .09361 | .027 | -.4406 | -.0327 |
|  |  |  |  | YL | .13000 | .09361 | .190 | -.0740 | .3340 |
|  |  |  |  | WL | .16000 | .09361 | .113 | -.0440 | .3640 |
|  |  |  |  | CK | .14000 | .09361 | .161 | -.0640 | .3440 |
|  |  | WL | dimension3 | RL | -.18333 | .09361 | .074 | -.3873 | .0206 |
|  |  |  |  | BL | -.39667^*^ | .09361 | .001 | -.6006 | -.1927 |
|  |  |  |  | YL | -.03000 | .09361 | .754 | -.2340 | .1740 |
|  |  |  |  | GL | -.16000 | .09361 | .113 | -.3640 | .0440 |
|  |  |  |  | CK | -.02000 | .09361 | .834 | -.2240 | .1840 |
|  |  | CK | dimension3 | RL | -.16333 | .09361 | .107 | -.3673 | .0406 |
|  |  |  |  | BL | -.37667^*^ | .09361 | .002 | -.5806 | -.1727 |
|  |  |  |  | YL | -.01000 | .09361 | .917 | -.2140 | .1940 |
|  |  |  |  | GL | -.14000 | .09361 | .161 | -.3440 | .0640 |
|  |  |  |  | WL | .02000 | .09361 | .834 | -.1840 | .2240 |

| **Total Flavonoid** | | | | |
| --- | --- | --- | --- | --- |
|  | Light Quality | N | Subset for alpha = 0.05 | |
|  |  |  | 1 | 2 |
| Duncan^a^ | WL | 3 | 1.5533 |  |
|  | CK | 3 | 1.5733 |  |
|  | YL | 3 | 1.5833 |  |
|  | GL | 3 | 1.7133 |  |
|  | RL | 3 | 1.7367 |  |
|  | BL | 3 |  | 1.9500 |
|  | Sig. |  | .099 | 1.000 |

**24. The ANOVA of Total Polyphenol**

| **ANOVA** | | | | | |
| --- | --- | --- | --- | --- | --- |
| Total Polyphenol | | | | | |
|  | Sum of squares | df | Mean square | F | Sig. |
| Between Groups | 4.654 | 5 | .931 | 5.304 | .008 |
| Within groups | 2.106 | 12 | .176 |  |  |
| Total | 6.761 | 17 |  |  |  |

| **Multiple Comparisons** | | | | | | | | | |
| --- | --- | --- | --- | --- | --- | --- | --- | --- | --- |
| Dependent Variable: Total Polyphenol | | | | | | | | | |
|  | (I) Light Quality | | (J)  Light Quality | | Mean Difference (I-J) | Std.Errer | Sig. | 95% Confidence Internal | |
|  |  |  |  |  |  |  |  | lower Bound | Upper Bound |
| LSD | dimension2 | RL | dimension3 | BL | -.39042 | .34206 | .276 | -1.1357 | .3549 |
|  |  |  |  | YL | .85417^*^ | .34206 | .028 | .1089 | 1.5995 |
|  |  |  |  | GL | 1.07958^*^ | .34206 | .008 | .3343 | 1.8249 |
|  |  |  |  | WL | .25292 | .34206 | .474 | -.4924 | .9982 |
|  |  |  |  | CK | .67917 | .34206 | .070 | -.0661 | 1.4245 |
|  |  | BL | dimension3 | RL | .39042 | .34206 | .276 | -.3549 | 1.1357 |
|  |  |  |  | YL | 1.24458^*^ | .34206 | .003 | .4993 | 1.9899 |
|  |  |  |  | GL | 1.47000^*^ | .34206 | .001 | .7247 | 2.2153 |
|  |  |  |  | WL | .64333 | .34206 | .084 | -.1020 | 1.3886 |
|  |  |  |  | CK | 1.06958^*^ | .34206 | .009 | .3243 | 1.8149 |
|  |  | YL | dimension3 | RL | -.85417^*^ | .34206 | .028 | -1.5995 | -.1089 |
|  |  |  |  | BL | -1.24458^*^ | .34206 | .003 | -1.9899 | -.4993 |
|  |  |  |  | GL | .22542 | .34206 | .522 | -.5199 | .9707 |
|  |  |  |  | WL | -.60125 | .34206 | .104 | -1.3465 | .1440 |
|  |  |  |  | CK | -.17500 | .34206 | .618 | -.9203 | .5703 |
|  |  | GL | dimension3 | RL | -1.07958^*^ | .34206 | .008 | -1.8249 | -.3343 |
|  |  |  |  | BL | -1.47000^*^ | .34206 | .001 | -2.2153 | -.7247 |
|  |  |  |  | YL | -.22542 | .34206 | .522 | -.9707 | .5199 |
|  |  |  |  | WL | -.82667^*^ | .34206 | .033 | -1.5720 | -.0814 |
|  |  |  |  | CK | -.40042 | .34206 | .264 | -1.1457 | .3449 |
|  |  | WL | dimension3 | RL | -.25292 | .34206 | .474 | -.9982 | .4924 |
|  |  |  |  | BL | -.64333 | .34206 | .084 | -1.3886 | .1020 |
|  |  |  |  | YL | .60125 | .34206 | .104 | -.1440 | 1.3465 |
|  |  |  |  | GL | .82667^*^ | .34206 | .033 | .0814 | 1.5720 |
|  |  |  |  | CK | .42625 | .34206 | .236 | -.3190 | 1.1715 |
|  |  | CK | dimension3 | RL | -.67917 | .34206 | .070 | -1.4245 | .0661 |
|  |  |  |  | BL | -1.06958^*^ | .34206 | .009 | -1.8149 | -.3243 |
|  |  |  |  | YL | .17500 | .34206 | .618 | -.5703 | .9203 |
|  |  |  |  | GL | .40042 | .34206 | .264 | -.3449 | 1.1457 |
|  |  |  |  | WL | -.42625 | .34206 | .236 | -1.1715 | .3190 |

| **Total Polyphenol** | | | | | | |
| --- | --- | --- | --- | --- | --- | --- |
|  | Light Quality | N | Subset for alpha = 0.05 | | | |
|  |  |  | 1 | 2 | 3 | 4 |
| Duncan^a^ | GL | 3 | 3.2963 |  |  |  |
|  | YL | 3 | 3.5217 | 3.5217 |  |  |
|  | CK | 3 | 3.6967 | 3.6967 | 3.6967 |  |
|  | WL | 3 |  | 4.1229 | 4.1229 | 4.1229 |
|  | RL | 3 |  |  | 4.3758 | 4.3758 |
|  | BL | 3 |  |  |  | 4.7662 |
|  | Sig. |  | .287 | .120 | .082 | .098 |
